# Chromosomal imbalance in the progression of high-risk non-muscle invasive bladder cancer

**DOI:** 10.1186/1471-2407-9-149

**Published:** 2009-05-16

**Authors:** Karsten Zieger, Carsten Wiuf, Klaus Møller-Ernst Jensen, Torben Falck Ørntoft, Lars Dyrskjøt

**Affiliations:** 1 Department of Molecular Medicine, Aarhus University Hospital Skejby, Aarhus N, Denmark; 2 Department of Urology, Aarhus University Hospital Skejby, Aarhus N, Denmark; 3 Bioinformatics Research Center, Aarhus University, Aarhus, Denmark

## Abstract

**Background:**

Non-muscle invasive bladder neoplasms with invasion of the *lamina propria *(stage T1) or high grade of dysplasia are at "high risk" of progression to life-threatening cancer. However, the individual course is difficult to predict. Chromosomal instability (CI) is associated with high tumor stage and grade, and possibly with the risk of progression.

**Methods:**

To investigate the relationship between CI and subsequent disease progression, we performed a case-control-study of 125 patients with "high-risk" non-muscle invasive bladder neoplasms, 67 with later disease progression, and 58 with no progression. Selection criteria were conservative (non-radical) resections and full prospective clinical follow-up (> 5 years). We investigated primary lesions in 59, and recurrent lesions in 66 cases.

We used Affymetrix GeneChip^® ^Mapping 10 K and 50 K SNP microarrays to evaluate genome wide chromosomal imbalance (loss-of-heterozygosity and DNA copy number changes) in 48 representative tumors. DNA copy number changes of 15 key instability regions were further investigated using QPCR in 101 tumors (including 25 tumors also analysed on 50 K SNP microarrays).

**Results:**

Chromosomal instability did not predict any higher risk of subsequent progression. Stage T1 and high-grade tumors had generally more unstable genomes than tumors of lower stage and grade (mostly non-primary tumors following a "high-risk" tumor). However, about 25% of the "high-risk" tumors had very few alterations. This was independent of subsequent progression. Recurrent lesions represent underlying field disease. A separate analysis of these lesions did neither reflect any difference in the risk of progression. Of specific chromosomal alterations, a possible association between loss of chromosome 8p11 and the risk of progression was found. However, the predictive value was limited by the heterogeneity of the changes.

**Conclusion:**

Chromosomal instability (CI) was associated with "high risk" tumors (stage T1 or high-grade), but did not predict subsequent progression. Recurrences after "high-risk" tumors had fewer chromosomal alterations, but there was no association with the risk of progression in this group either. Thus, the prediction of progression of "high risk" non-muscle invasive bladder tumors using chromosomal changes is difficult. Loss of chromosome 8p11 may play a role in the progression process. About 25% of the "high risk" tumors were chromosomal stable.

## Background

The individual course of non-muscle invasive bladder neoplasm is difficult to predict. In particular, tumors with invasion of the *lamina propria *(stage T1) or high grade of dysplasia have a 30–60% risk of progression to muscle invasive, life-threatening cancer [1,2]. A transurethral, bladder-sparing approach is often chosen to cure non-muscle invasive tumors. However, recurrence is common [2,3]. Remnants of tumor tissue due to incomplete resection would be the source of true tumor recurrence at the original site. New tumor occurrences at distant sites, however, are owed to cancer precursors in the mucosa – so-called field disease – a very common condition in bladder cancer. Multifocality or concomitant *carcinoma in situ *(CIS), a highly dysplastic flat precursor lesion, may be indicators of field disease. The evaluation of the malignant potential of field disease is of particular clinical interest.

The assessment of molecular changes may improve this evaluation. The hitherto best known molecular event predictive of advanced malignant development is the loss of p53 function, which is found in the majority of progressed bladder cancers and, notably, in CIS [4,5]. Several mechanisms may lead to loss of p53 function, e.g. gene mutation or loss, transcriptional downregulation and enhanced degradation. P53 is part of the complex DNA damage control system, which surveys the integrity of the genome. Dysfunction of this system leads to chromosomal instability (CI), a key event of malignant tumor development [6]. CI is easier to assess and to interpret than loss of p53 function, and is currently employed for the detection of tumor-cells in the urine using fluorescence in-situ hybridisation and microsatellite analysis [7]. Primary tumors and subsequent recurrences are usually of clonal origin. Evaluation of the CI of recurrences may thus gain insight into the malignant potential of field changes.

CI can possibly predict the risk of progression. Richter et al [8] studied 54 stage T1 bladder cancers by conventional comparative genomic hybridisation (CGH). This study suggested that multiple chromosomal losses, as well as copy number changes at certain genomic regions, herald a shorter progression free survival and thus a worse prognosis [8]. Recent advances in microarray technology made it possible to screen the genome for chromosomal imbalances (= loss of heterozygosity (LOH) and/or DNA copy number (CN) alterations) with high resolution [9-13]. Single nucleotide polymorphism (SNP) microarrays offer the opportunity to study LOH and CN changes simultaneously [14]. We previously studied chromosomal instability during the progression of bladder cancer using SNP microarrays [10]. In this paper, we aimed at a genome-wide investigation of inherent chromosomal alterations related to the risk of progression in bladder cancer. We present an extended material of 48 mostly high-risk non-muscle invasive bladder cancers with special reference to subsequent progression. Furthermore, we performed CN analyses of 15 most frequently changed gene-loci using quantitative polymerase chain reaction (QPCR) in 77 independent test tumors for validating the association of the changes with the risk of progression of non-muscle invasive bladder cancer.

## Methods

### Study design, patient selection and follow-up

This case-control study was based on a tissue bank with samples from consecutive bladder cancer patients, treated at our institution since 1994. Patients were followed prospectively. The study was approved by the scientific ethical committee of the county of Aarhus, and all patients gave their informed written consent.

Bladder tumor specimens were cleaved immediately after transurethral resection. One half was analysed by conventional histopathology, the rest snap-frozen in liquid nitrogen and stored at -80°C. Tumors were staged according to TNM and graded according to the Bergkvist-classification [15] on a routine base.

We previously analysed tumors from 19 patients from this cohort using Affymetrix^® ^Early Access GeneChip^® ^Mapping 10 K SNP microarrays (Affymetrix, Santa Clara, CA) [10,16]. To study the impact of chromosomal alterations on the risk of future disease progression, we extended this dataset by retrospectively selecting further 106 patients from the cohort to total 125 patients. Selection criteria, besides the availability of material, were: non-muscle invasive disease (no previous muscle invasion), a full clinical follow-up without radical treatment (at least five years), and a disease course with "high-risk" tumors (stage T1 or grade 3) or progression (to muscle invasion or metastasis). See Additional File 1 for clinical data of patients and tumors.

Primary tumors from 59 patients were available. Most of these tumors were "high-risk" (stage T1 or grade 3); however, we included 4 primary stage Ta grade 2 tumors with later progression ("high-risk in retrospect") (Table 1). The aim was to investigate inherent chromosomal alterations related to the risk of progression in bladder cancer.

**Table 1:** Stage and grade distribution of the study material of 125 non-muscle invasive bladder cancers.

		Tumors with later progression					Tumors with no progression				Totals
Stage/Grade		TaG2*	TaG2	TaG3/CIS	T1G2	T1G3	TaG2	TaG3/CIS	T1G2	T1G3	

Primary tumors	SNP microarray	1			1	10				10	22
	QPCR only	3		1	1	10			6	16	37
Total primary tumors		4		1	2	20			6	26	59
Secondary tumors	SNP microarray	1	2	6		6	2	4	1	4	26
	QPCR only	2	4	9		10	4	5	2	4	40
Total secondary tumors		3	6	15	0	16	6	9	3	8	66
Stage distribution (totals)		7	6	16	2	36	6	9	6	26	125
Mean age (range)		72.4 (54–85)					68.4 (46–83)				
Gender	Male	54					44				98
	Female	13					14				27
Totals		67					58				125

Distribution into primary and secondary tumors, as well as the type of analysis performed, is indicated. Seven low-risk cases with later progression were included and marked with asterisk*. All other tumors were high-risk, or recurrence after high-risk tumors. Detailed clinical information about all individual cases including primary and current stage, grade, treatment, CIS-status, progression free survival, metastases, overall survival and causes of death are listed in Additional File 1.

To investigate whether these changes also were present in recurrences, i.e. in field disease, we examined non-primary tumors from 66 different patients. All but three were followed for a previous "high-risk" tumor, but fifteen of the non-primary tumors were "low-risk" themselves (stage Ta grade 2) (Table 1). Non-primary and primary tumors of this study were independent, i.e. from different patients.

Preselected site biopsies were taken to diagnose concomitant CIS. No adjuvant intravesical chemotherapy was given. Patients were followed prospectively in a routine schedule. Disease recurrences were treated in a standardized fashion. An induction course of intravesical *Bacille Calmette-Guerin *instillations was applied in 30 cases (CIS, multiple tumors, high recurrence rate), but was not standard treatment at that time. The progression-free survival time was censored at the time of the last control cystoscopy with no evidence of disease. Detailed clinical data are available in Additional File 1.

### DNA-purification and SNP microarray analysis

DNA from frozen tumor tissue and corresponding genomic DNA from blood leucocytes was extracted and purified using a standard DNA-extraction kit (Gentra, Minneapolis, MN). Tumor tissue was trimmed in the microscope to ensure at least 75% tumor cells. Previously purified DNA (kept at -20 to -80°C) was used in some cases, having been extracted using various methods, including phenol and ethanol extraction. Concentration and OD ratios were determined using a spectrophotometer.

For high-resolution genome screening, we expanded the previously analysed 10 K SNP microarray dataset of 19 tumors [16] with further 29 tumors to total 48 tumors. A selection was made to achieve a homogeneous dataset with respect to the distribution of primary/recurrent, stage T1/stage Ta, and progression/no progression; otherwise it was random. Due to technological progress, the Early Access 10 K SNP microarray was no longer available; instead we used contemporary Affymetrix Xba GeneChip^® ^Mapping 50 K SNP microarrays. Analyses of tumor and corresponding blood-DNA were performed as prescribed by the manufacturer. The method was similar for both array types, however, the analyses were performed independently of each other. In brief, 250 ng of DNA was digested using Xba I restriction enzyme, ligated to an Xba adaptor and amplified by PCR using adaptor-specific primers. After purification, 40 μg of the PCR products were fragmented using DNase I, labelled, and hybridised to the microarrays at 48°C for 18 hours. After hybridisation, arrays were washed 2 times, stained with streptavidin-phycoerythin, washed, linked to biotinylated anti-streptavidin antibodies, stained again, and finally washed, followed by reading in a laser scanner. We used an automated Fluidics^® ^station and the GCOS^® ^software provided by the manufacturer.

#### LOH-analysis

10 K and 50 K SNP array data were normalized and analysed independently, using empirical means and standard deviations (sd) of 113 [10 K SNP arrays] and 67 [50 K SNP arrays] normal DNA samples extracted from blood leucocytes, respectively, as described [16]. To obtain allelic calls for tumor DNA and the corresponding normal genomic DNA, we used the GDAS^® ^genotyping software supplied by the array manufacturer. Subsequently, a Hidden Markov Model was applied to infer the probability *a*_*ij *_of allelic imbalance/loss of heterozygosity (LOH) for each SNP *j *on each tumor array *i *compared to the corresponding array of normal DNA, using the DChip software. For details, see http://biosun1.harvard.edu/complab/dchip/snp.htm, and [17]. For the calculation of the fraction of the genome altered (FGA [LOH]), a probability *a *> 0.2 was considered as LOH for both array types.

#### Copy number analysis

10 K and 50 K SNP array data were normalized and analysed separately, as mentioned above. The arrays were normalized and signal values for the individual SNPs were extracted using the DChip software [14]. Briefly, an invariant set normalization method was applied to normalize all arrays of one type at the probe intensity level to a baseline array. After normalization, the perfect match/mismatch difference model was applied to extract a single signal value for each SNP on each array.

Data was then normalized SNP-wise to have mean zero and SD one, using the empirical mean and SD from the respective normal arrays, z_*ij *_= (y_*ij *_- m_*j*_)/sd_*j*_, where y_*ij *_is the signal value for array *i *and SNP *j*, m_*j *_is the mean of SNP *j *and sd_*j *_the SD of SNP *j*. Data z_*ij *_was further smoothened over *k *SNPs to obtain *x*_*ij *_= *Σ z*_*ik*_/*k*, where the sum is over *k *= *j *- *K*,...,*j*+*K*. Here *K *= 5 for both 10 K and 50 K arrays. For the calculation of the fraction of the genome altered (FGA [CN]), signal values *x *higher than 3 or lower than -3 (mean +/-3 sd, corresponding to probability *p *of 1.degree's error < 0.01) were considered copy number imbalanced.

#### Creation of common SNP dataset

A total of 6,400 common probesets were identified on both microarrays, using the annotation-files provided by the array-manufacturer. Data *a *(LOH probability) and *x *(standardised signal value) for these SNPs were extracted from both the 10 K and 50 K datasets and assigned to unique reference-SNP-IDs http://www.ncbi.nlm.nih.gov/SNP. The combined dataset included data from 48 patients, 19 analysed on 10 K arrays and 29 analysed on 50 K arrays. LOH data was missing for one 50 K array (#8-3) because of unmatched blood-DNA, and signal intensity data were lost for two 10 K arrays (#1013-1 and #1033-2) after extraction of the allelic calls.

The overall median FGA (both FGA [LOH] and FGA [CN]) was 5%, which was chosen as the cut-off value in the survival analyses.

#### Test for differences in chromosome alterations between progressing and non-progressing tumors

The average of x_*ij *_was calculated for tumors in the 2 groups (progressors and non-progressors) for each SNP, and the difference d_*j *_between the two averages was found. High and low values of d_*j *_were indicative of group differences. Low values indicated that group 1 had higher mean than group 2, and high values that group 2 had higher mean. Long segments of SNPs with high (or low) values of d_*j *_were more likely to be due to true differences; thus the segment lengths were also calculated. Significance of the observed statistics was calculated using a permutation test: New groups of the same sizes were formed by permuting group labels 50,000 times and the number of times (in %) a value higher (or lower) was observed, was calculated. This test was performed using the combined dataset (see Additional Files 2 and 3) and using the 50 K dataset alone, because of the higher resolution of this dataset (see Additional Files 4 and 5).

### Copy number analysis using QPCR

Fifteen unstable chromosomal regions were identified using the SNP microarray data (see Additional Files 2, 3, 4 and 5). Within these regions, we selected 15 gene loci of documented or putative bladder cancer relevance (Table 2). To determine relative DNA copy number changes of these loci, we performed QPCR using an ABI Prism 7500^® ^Real time PCR cycler (Applied Biosystems, Foster City, CA) with standard QPCR conditions according to the manufacturer's instructions (all analyses were performed in triplicate). Primer sequences are listed in Additional File 6. Standard curves were constructed using pooled normal DNA from blood.

**Table 2:** Selected gene loci, gene IDs, cytoband and genomic annotation of the amplicons used for QPCR validation.

Gene	Gene-ID	Cytoband	Amplicon position (NCBI Build 35.1 [hg17])	Amplicon position (hg18)
*S100A8*	6279	1q21	chr1:150,175,956–150,176,071	chr1:151,629,507–151,629,622
*FN1*	2335	2q34	chr2:216,112,380–216,112,466	chr2:215,995,119–215,995,205
*RAF1*	5894	3p25	chr3:12,635,039–12,635,148	Identical
*DAB2*	1601	5p13	chr5:39,418,493–39,418,602	identical
*CSPG2*	1462	5q14.3	chr5:82,821,732–82,821,832	identical
*E2F3*	1871	6p22	chr6:20,598,410–20,598,516	identical
*SFRP1*	6422	8p12-p11.1	chr8:41,285,606–41,285,686	identical
*TUSC3*	7991	8p22	chr8:15,524,970–15,525,081	identical
*EDD1*	51366	8q22	chr8:103,366,989–103,367,080	identical
*MYC*	4609	8q24.12-q24.13	chr8:128,822,158–128,822,287	identical
*CDKN2A*	1029	9p21	chr9:21,957,845–21,957,973	identical
*KLF4*	9314	9q31	chr9:107,331,117–107,331,240	chr9:109,291,383–109,291,506
*MDM2*	4193	12q14.3-q15	chr12:67,519,751–67,519,858	Identical
*RB1*	5925	13q14.2	chr13:47,853,426–47,853,564	Identical
*TP53*	7157	17p13.1	chr17:7,520,091–7,520,236	Identical

Loci were selected based on the frequency of alterations in the total SNP material, and on bladder cancer relevance according to literature and our own gene expression analyses [22]. Loci are not referring to the (insignificant) relative changes characterizing progressing tumors listed on Table 3, except *SFRP1 *(8p11). Primer sets leading to amplicons of 80–120 basepairs were designed using the Primer3 software http://frodo.wi.mit.edu/cgi-bin/primer3/primer3.cgi. The QPCR primer sequences are listed in Additional File 6.

We successfully analysed 101 tumors, including 25 tumors analysed on 50 K SNP microarrays. The obtained quantity values were standardised to mean one by division with the median quantity value of the 15 examined probes (the median value was considered to be most likely to represent a normal copy number). Results were log transformed. Normal variation was estimated using tumors that were stable on 50 K microarrays (FGA < 0.05) as standard. Quantity values exceeding two times the normal variation were considered copy number imbalanced. We used the STATA 8.0 statistical software package (StataCorp LP, College Station, TX).

## Results

In total, 67 patients (54%) suffered disease progression, which was defined as occurrence of muscle invasion (stage T2-4) or metastatic disease (stage N2-3 or stage M1). The high progression rate is due to the study design (exclusion of radically treated patients and patients with no progression but incomplete follow-up). Fifty-eight patients showed no evidence of progression. Follow-up was median 80 months. The median time to progression was 17 months. Nine patients had longer than five years progression-free survival, with late progression. We found CIS concomitant to 38 tumors (31%), with equal distribution between progressors and non-progressors in this high-risk material (Table 1).

### SNP microarrays (n = 48): Fraction of the genome altered (FGA)

**Figure 1:**
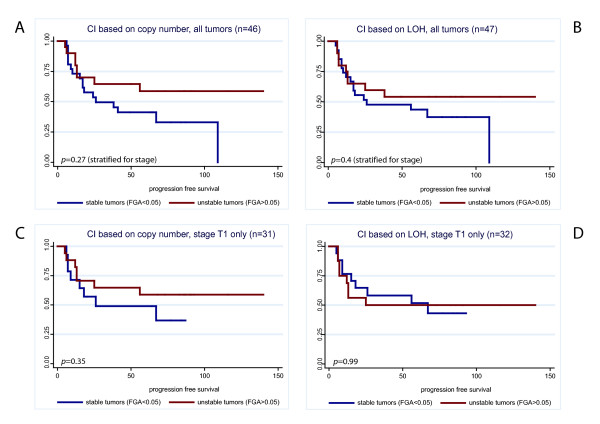
**Kaplan Meier estimates of progression-free survival according to chromosomal instability (CI)**. Tumors are divided in stable and unstable using a cut-off for the fraction of the genome altered (FGA) > 0.05. Here, the influence of the time to progression, not the relative size of the FGA is analysed. Logrank-tests for differences between progressing and non-progressing tumors showed no significant difference. **A: **all tumors, CI based on copy number changes (n = 46). **B: **all tumors, CI based on LOH (n = 47). **C: **Stage T1 tumors only, CI based on copy number changes (n = 32). **D: **Stage T1 tumors only, CI based on LOH (n = 33).

**Figure 2:**
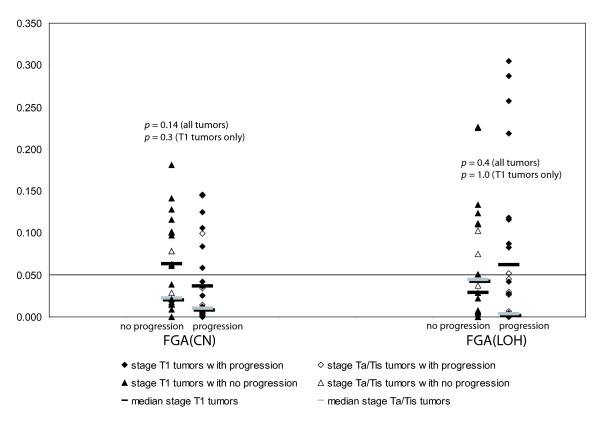
**The fraction of the genome altered (FGA) against the clinical course (progression/no progression), separately for copy number changes (left) and LOH (right)**. Non-progressing tumors in triangles, progressing in diamonds. Solid symbols indicate stage T1 tumors. Ranksum-tests for difference in FGA between progressing and non-progressing tumors for all tumors (n = 48) and for stage T1 tumors only (n = 33) showed no significant difference. Here, the relative size of the FGA, not the influence of the time to progression is analysed.

The dataset was composed of 22 primary tumors (hereof 21 stage T1), and 26 recurrent tumors (hereof 15 stage T1). 27 patients had progressing disease, while 21 had no progression (see Additional File 1 for details). The probability of later progression was not associated with the fraction of the genome altered (FGA). Figure 1 illustrates the progression-free survival, dependent on whether the examined tumor was stable or unstable. Results of all tumors (corrected for stage) and separately for stage T1 tumors are shown. Figure 2 shows the relative significance of the FGA and its variation, dependent on whether later progression occurred or not. The FGA [CN] and FGA [LOH] for all individual tumors is listed in Additional File 7.

In primary tumors separately (n = 22, hereof 21 stage T1) we found no difference between tumors with and with no later progression (*p *= 0.62 and *p *= 0.2, for FGA(LOH) and FGA(CN), respectively; Mann-Whitney ranksum tests). Recurrent lesions (n = 26, stage T1: n = 15), representing underlying field disease, even had a slight tendency towards more stable tumors among those with later progression (*p *= 0.08 and *p *= 0.52; corrected for T-stage). Equivalent results were obtained using Kaplan-Meier-estimates of progression-free survival time (stable vs. unstable). Cox' regression analyses, taking into account both the time to progression and the relative significance of FGA (in categories), did neither reveal any significant differences.

In general, the FGA was significantly higher for stage T1 tumors (*p *= 0.004 and *p *= 0.0005) and grade 3 tumors (*p *= 0.03 and *p *= 0.003) compared to stage Ta and grade 2 tumors, respectively. The FGA was also positively correlated to the occurrence of CIS. Notably, eleven tumors (23%) had very few alterations (FGA < 0.01). Interestingly, seven of these tumors were stage T1, and the disease progressed in seven of these cases (four stage T1 tumors).

### 3.2. Test for differences in chromosomal alterations related to subsequent progression

This test was performed to reveal specific chromosomal areas related to subsequent progression. However, the tumors showed wide chromosomal heterogeneity, which resulted in a considerable risk of detecting "significance by chance", supposed the relatively low number of samples (n = 48) compared to the number of variables (n = 6,400). We employed the fact that all SNPs are mutually dependent through their mapped positions: a number of neighbouring "significant" SNPs altered in the same way were more likely to represent true changes (segment analysis). Genome-wide differences are shown in Additional Files 2 and 3 (Combined dataset, CN and LOH differences, respectively), and with the higher resolution of the 50 K dataset in Additional Files 4 and 5.

Using a combined approach of LOH and copy number analysis with restriction to changes more pronounced in progressing tumors (and thus independent of stage), we identified some areas which may play a role in the progression of bladder cancer (Table 3). Most prominent was loss/LOH of chromosome 8p11-p12 (35–43 Mb). To reveal statistic significance of these segments, we performed a permutation analysis. However, none of the identified areas was significant in segment analysis (Table 3).

**Table 3:** Candidate areas with chromosomal alterations characterizing non-muscle invasive bladder cancers with subsequent progression.

Chromosome	Cytoband	Physical position (hg17/NCBI build 35.1)	chromosomal changes of progressing tumors	Candidate genes (selection)	*p *(single SNPs)	*p *(segment)
1	p31.1-p22.3	82–86 Mb	CN gain	BCL10	0.003	n.s.
2	q33.3-q34	208–213 Mb	CN gain	CREB1, MAP2	0.001	0.05
3	p21-p14.3	42–64 Mb	CN gain	CCR-cluster	0.01	n.s.
4	p13	40–45 Mb	CN loss/LOH		0.02	n.s.
6	q15-q23.2	89–133 Mb	CN loss/LOH	TPD52L1	0.01	n.s.
8	p12-p11.21	33 M–43 Mb	CN loss/LOH	SFRP1, TACC1, FGFR1	0.01	n.s.
10	p15.1-p14	3.5 M–13 Mb	CN gain	GATA3, NET1, PKCQ, IL15RA	0.005	n.s.
10	p11	30–36 Mb	CN gain		0.005	n.s.
15	q25.3	84–85 Mb	CN loss		0.01	n.s.

SNP microarray analysis (significance *p *= probability of detecting difference between progressing and non-progressing tumors by chance, permutation analysis). A survey of the entire genome is possible in Additional Files 2, 3, 4 and 5.

### 3.3. Copy number analysis of 15 selected gene loci using QPCR (101 tumors, Figure 3)

**Figure 3:**
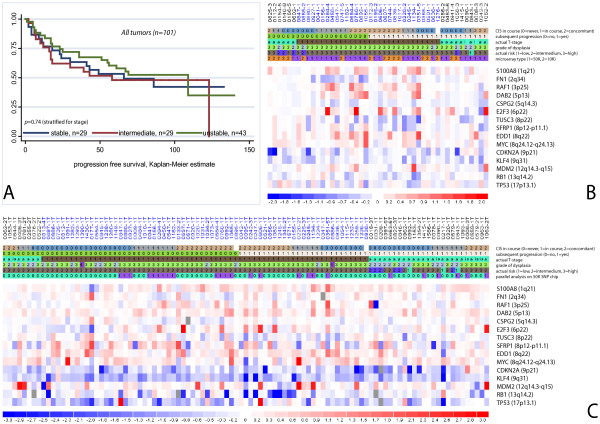
**Copy number analysis of 15 marker regions (see Table 2)**. **A: **Kaplan Meier estimates of progression free survival according to number of copy-number imbalanced marker regions in QPCR analysis. Stable (0–2 changes); intermediate (3–4 changes), unstable (> 4 changes). **B: **Copy number alterations (log2 ratios) of candidate regions determined by SNP microarrays (SNPs flanking the marker regions). Tumors in columns, marker regions in rows. Top row indicates patient and tumor ID, bars below refer to tumor characteristics. Non-progressors to the left, progressors to the right; stage T1 tumors in the middle, stage Ta tumors on the flanks. Tumors are further sorted according to their CIS status: Tumors with concomitant CIS in the very center and on the very verges, tumors with later or no CIS in between. Furthermore, the grade of dysplasia (Bergkvist[15]), the actual clinical risk (including previous history)[3] and the used microarray (1 = 50 K, 2 = 10 K) are indicated. Data area: Red: Copy number gain. Blue: Copy number loss. **C: **Copy number analysis of the same candidate regions by QPCR of 101 tumors, hereof 77 independent tumors. Tumors are sorted in the same way as in **B**. A survey of the number of altered regions is provided in Additional File 7.

A semi quantitative illustration of the results in all tumors is given on Figure 3. We confirmed that stage T1 tumors had significantly more alterations than stage Ta tumors (*p *= 0.008, Mann-Whitney-test). In congruence with the microarray results, there was no correlation between the number of alterations and subsequent progression. QPCR data showed good association with the data obtained from 50 K microarrays (*r *= 0.67). However, no correlation between any of the markers and the risk of progression was found, including the *SFRP1 *marker (8p11-p12). Neither did we find significant influence of any confounder studied, including age, gender, multiplicity, primary/secondary, concomitant CIS, and BCG-treatment, in multivariate analyses. The number of changed markers for all individual tumors is listed in Additional File 7.

## Discussion

The aim of the present study was to investigate inherent chromosomal changes related to the risk of progression in bladder cancer, as well as chromosomal instability (CI)/fraction of the genome altered (FGA) as a prognostic marker. A previous CGH study of 54 stage T1 tumors suggested a shorter progression free survival of tumors with multiple copy number losses (CI) or alterations at certain regions [8]. We utilised SNP microarrays to perform high-resolution genome-wide combined LOH and copy number screening of 21 primary stage T1 tumors. We demonstrated frequent chromosomal changes in most of these tumors. However, we were not able to show any association between chromosomal changes and subsequent progression. One explanation may be that stage T1 tumors can be completely resected. Under this condition, the future course is not determined by the malignant potential of the resected tumor, but the residual tumor cells in the bladder.

Recurrent tumors are usually the result of the spread of a single tumor clone in the urothelium. Only a minority has been shown to be of different clonal origin, i.e. new primary tumors [18]. We analysed 26 recurrent tumors, to determine whether residual tumor cells in the bladder already harbour changes related to the risk of progression, because these changes should be present in all recurrences as well. Our results demonstrated less frequent chromosomal alterations in non-invasive recurrences (stage Ta), even if the primary lesion was stage T1, and the disease later progressed. Recurrent stage T1 tumors had as much alterations as the primary stage T1 tumors; however, this was again independent of later progression. These results indicated that the precursor lesions of recurrent tumors – also those with later progression – had relatively few alterations, i.e. they were apparently chromosomal stable. The frequent alterations seen in stage T1 (and muscle-invasive) tumors were then the results of (more or less) stochastic events during the development of the individual tumor, and not inherent characteristics of field changes with high malignant potential. These findings are well in-line with the "field-first-tumor-later" concept recently developed by Höglund [19], according to which recurring tumors originate from a shared field of premalignant cells (with relatively few chromosomal changes) and not from previous overt tumors. Consequently, it was impossible to predict the future course of a disease from chromosomal changes of a marker lesion, given that this lesion was completely resected. Notably, about 25% of the tumors showed virtually no CI. This was independent of stage, concomitant CIS, and future progression. In conclusion, with the huge variation of chromosomal changes demonstrated, chromosomal alterations are unlikely to play a role as a predictive instrument in non-muscle invasive bladder cancer – at least with the resolution provided here, which is higher than in the previous report [8].

The same considerations also apply with respect to specific chromosomal alterations. The paper of Richter et al. [8] suggested several specific chromosomal changes that may have significance for the malignant development independent of general CI. Unfortunately, we were not able to confirm these results. The identification of significant areas among many possible areas is challenging, given the variation of chromosomal changes observed. If the number of possible areas (here: SNPs) by far exceeds the number of samples, conventional statistical methods do not perform well. Correction for multiple comparisons is mandatory. In our segment analysis, we employed the dependency of the SNPs on their mapped positions to reduce the number of variables from 6,400 (SNPs) to 22 (chromosomes). This permutation analysis reveals the probability of finding a segment of significant SNPs of a given length on a given chromosome by chance. However, this analysis must fail to identify small significant segments, particularly on chromosomes with common large-scale alterations, e.g. chromosome 8.

We had the intention to validate the predictive potential of specific chromosomal areas using QPCR-based copy-number analysis. Since no significant areas could be documented (Table 3), we decided to evaluate general CI using areas with frequent instability (see Additional Files 2, 3, 4 and 5) and possible bladder cancer relevance, according to the literature (including [8]) and our own gene expression analyses. We did not find any correlation between the risk of progression and any of the chosen markers, or with the number of changed markers/CI. These results were in line with the SNP-data. However, the area on chromosome 8p11 deserves special attention. Not only did we pinpoint the area as significant in SNP analyses (Table 3), it has also previously been stressed as being very unstable not only in bladder cancer, but in many other solid cancers [20]. The area has been reported to be frequently involved in chromosomal rearrangements [20]. Candidate genes in this region include *SFRP1*. Chromosomal loss and reduced expression of the mRNA and the SFRP1 protein, an antagonist of Wnt-signalling, have been reported by several other groups to be associated with bladder cancer progression [21], and we found the same in our own gene expression profiles [22]. In QPCR analysis, the *SFRP1 *marker was very unstable; however, this was not related to progression free survival. The clinical significance of this region still remains to be elucidated.

We adjusted for the grade of dysplasia, an important risk factor for the clinical risk estimation, using the Bergkvist grading system under routine conditions [15]. This system has been in routine use all over Scandinavia for many years, but is uncommon in the rest of the world. It can be translated into the 1999 WHO/ISUP grading system [23], according to which most of our tumors would be classified as "high grade". A pathological review of the slides would have been optimal, but was not performed. However, we considered the grading system of minor importance, as long as the analysis is adjusted for this factor.

## Conclusion

Chromosomal instability did not characterise tumors with subsequent progression. Our results suggest that chromosomal instability develops during the progression process, and is rare in precursor lesions, as indicated by the less pronounced chromosomal changes in recurrent tumors. Chromosomal alterations, particularly losses, of chromsome 8p11 (34–43 Mb) may be present in field disease, and may have a role in its malignant development.

## Abbreviations

(CIS): Carcinoma in situ of the urinary bladder; (DNA):desoxyribonucleic acid; (SNP): single nucleotide polymorphism; (CI): chromosomal instability; (CN): DNA copy number; (LOH): loss of heterozygosity; (FGA): fraction of the genome altered; (QPCR): quantitative polymerase chain reaction.

## Competing interests

The authors declare that they have no competing interests.

## Authors' contributions

KZ reviewed the patients' history and pathological data, performed sample selection and retrieval, carried out the molecular analyses, performed data analysis and part of the statistical analyses, interpretation of data, and drafted the manuscript. CW developed and carried out the procession of the microarray data and the statistical analyses. KMEJ was responsible for the sampling of tissue, information of patients, and the clinical database. TFØ participated in the conception and design of the study, and critically revised the manuscript. LD helped with the analysis and interpretation of the data, and helped to draft the manuscript.

## Pre-publication history

The pre-publication history for this paper can be accessed here:

http://www.biomedcentral.com/1471-2407/9/149/prepub

## Supplementary Material

Additional file 1 **Clinical data of the patients in the study**. Including age and gender, stage and grade of the primary and (if different) analysed tumors, supplementary treatment (BCG), CIS-status, length of follow-up/progression-free survival, causes of death and overall survival. Click here for file

Additional file 2 **Genome-wide copy number differences, 50 K+10 K data (6.4 K resolution)**. Graphical illustration of genome-wide copy number differences between tumors with and with no subsequent progression. Compiled results of all SNP microarray data (n = 46) with a resolution of 6.4 K SNPs. Click here for file

Additional file 3 **Genome-wide differences in probability of LOH, 50 K+10 K data**. Graphical illustration of genome-wide differences in probability of LOH between tumors with and with no subsequent progression. Compiled results of all SNP microarray data (n = 47). Click here for file

Additional file 4 **Genome-wide copy number differences, 50 K data only (41 K resolution)**. Graphical illustration of genome-wide copy number differences between tumors with and with no subsequent progression. 50 K SNP microarray data only (n = 29). Click here for file

Additional file 5 **Genome-wide differences in probability of LOH, 50 K data only**. Graphical illustration of genome-wide differences in probability of LOH between tumors with and with no subsequent progression. 50 K SNP microarray data only (n = 28). Click here for file

Additional file 6 Selected gene loci, primer sequences and genomic annotation of the amplicons used for QPCR validation. Click here for file

Additional file 7 **Chromosomal imbalance of the analysed tumors**. Contains detailed data of the fractions of the genome altered (FGA), specified separately for copy number and LOH changes, as well as the number of QPCR copy number alterations Click here for file

## References

[B1] Herr HW Natural history of superficial bladder tumors: 10- to 20-year follow-up of treated patients World J Urol 1997 15 84 88 10.1007/BF02201977 9144896

[B2] Zieger K Olsen PR Wolf H Hojgaard K Long term follow-up of superficial invasive bladder carcinoma with or without concomitant epithelial atypia – recurrence and progression Scand J Urol Nephrol 2002 36 52 59 10.1080/003655902317259373 12002359

[B3] Sylvester RJ Meijden AP van der Oosterlinck W Witjes JA Bouffioux C Denis L Predicting recurrence and progression in individual patients with stage Ta T1 bladder cancer using EORTC risk tables: a combined analysis of 2596 patients from seven EORTC trials Eur Urol 2006 49 466 5 10.1016/j.eururo.2005.12.031 16442208

[B4] Spruck CH III Ohneseit PF Gonzalez-Zulueta M Esrig D Miyao N Tsai YC Two molecular pathways to transitional cell carcinoma of the bladder Cancer Res 1994 54 784 788 8306342

[B5] Hartmann A Schlake G Zaak D Hungerhuber E Hofstetter A Hofstaedter F Occurrence of chromosome 9 and p53 alterations in multifocal dysplasia and carcinoma in situ of human urinary bladder Cancer Res 2002 62 809 818 11830537

[B6] Catto JW Meuth M Hamdy FC Genetic instability and transitional cell carcinoma of the bladder BJU Int 2004 93 19 24 10.1111/j.1464-410X.2004.04548.x 14678361

[B7] van Rhijn BW Poel HG van der Kwast TH van der Urine markers for bladder cancer surveillance: a systematic review Eur Urol 2005 47 736 748 10.1016/j.eururo.2005.03.014 15925067

[B8] Richter J Wagner U Schraml P Maurer R Alund G Knonagel H Chromosomal imbalances are associated with a high risk of progression in early invasive (pT1) urinary bladder cancer Cancer Res 1999 59 5687 5691 10582685

[B9] Hoque MO Lee CC Cairns P Schoenberg M Sidransky D Genome-wide genetic characterization of bladder cancer: a comparison of high-density single-nucleotide polymorphism arrays and PCR-based microsatellite analysis Cancer Res 2003 63 2216 2222 12727842

[B10] Koed K Wiuf C Christensen LL Wikman FP Zieger K Moller K High-density single nucleotide polymorphism array defines novel stage and location-dependent allelic imbalances in human bladder tumors Cancer Res 2005 65 34 45 15665277

[B11] Blaveri E Brewer JL Roydasgupta R Fridlyand J DeVries S Koppie T Bladder cancer stage and outcome by array-based comparative genomic hybridization Clin Cancer Res 2005 11 7012 7022 10.1158/1078-0432.CCR-05-0177 16203795

[B12] Veltman JA Fridlyand J Pejavar S Olshen AB Korkola JE DeVries S Array-based comparative genomic hybridization for genome-wide screening of DNA copy number in bladder tumors Cancer Res 2003 63 2872 2880 12782593

[B13] Hurst CD Fiegler H Carr P Williams S Carter NP Knowles MA High-resolution analysis of genomic copy number alterations in bladder cancer by microarray-based comparative genomic hybridization Oncogene 2004 23 2250 2263 10.1038/sj.onc.1207260 14968109

[B14] Zhao X Li C Paez JG Chin K Janne PA Chen TH An integrated view of copy number and allelic alterations in the cancer genome using single nucleotide polymorphism arrays Cancer Res 2004 64 3060 3071 10.1158/0008-5472.CAN-03-3308 15126342

[B15] Bergkvist A Ljungqvist A Moberger G Classification of bladder tumours based on the cellular pattern. Preliminary report of a clinical-pathological study of 300 cases with a minimum follow-up of eight years Acta Chir Scand 1965 130 371 378 5842510

[B16] Zieger K Dyrskjot L Wiuf C Jensen JL Andersen CL Jensen KM Role of activating fibroblast growth factor receptor 3 mutations in the development of bladder tumors Clin Cancer Res 2005 11 7709 7719 10.1158/1078-0432.CCR-05-1130 16278391

[B17] Lin M Wei LJ Sellers WR Lieberfarb M Wong WH Li C dChipSNP: significance curve and clustering of SNP-array-based loss-of-heterozygosity data Bioinformatics 2004 20 1233 1240 10.1093/bioinformatics/bth069 14871870

[B18] Hafner C Knuechel R Stoehr R Hartmann A Clonality of multifocal urothelial carcinomas: 10 years of molecular genetic studies Int J Cancer 2002 101 1 6 10.1002/ijc.10544 12209580

[B19] Hoglund M On the origin of syn- and metachronous urothelial carcinomas Eur Urol 2007 51 1185 1193 10.1016/j.eururo.2006.11.025 17123702

[B20] Adams J Williams SV Aveyard JS Knowles MA Loss of heterozygosity analysis and DNA copy number measurement on 8p in bladder cancer reveals two mechanisms of allelic loss Cancer Res 2005 65 66 75 15665280

[B21] Stoehr R Wissmann C Suzuki H Knuechel R Krieg RC Klopocki E Deletions of chromosome 8p and loss of sFRP1 expression are progression markers of papillary bladder cancer Lab Invest 2004 84 465 478 10.1038/labinvest.3700068 14968126

[B22] Dyrskjot L Zieger K Kruhoffer M Thykjaer T Jensen JL Primdahl H A molecular signature in superficial bladder carcinoma predicts clinical outcome Clin Cancer Res 2005 11 4029 4036 10.1158/1078-0432.CCR-04-2095 15930337

[B23] Busch C Algaba F The WHO/ISUP 1998 and WHO 1999 systems for malignancy grading of bladder cancer. Scientific foundation and translation to one another and previous systems Virchows Arch 2002 441 105 108 10.1007/s00428-002-0633-x 12189498

