# Supplementary material for: Chromosomal imbalance in the progression of high-risk non-muscle invasive bladder cancer
Source: BMC Cancer. 2009 May 16;9:149. doi: 10.1186/1471-2407-9-149 (PMC2696467; doi:10.1186/1471-2407-9-149)

Additional File 2

Genome-wide graphical presentation and test for relative differences in chromosomal alterations between tumors with and without subsequent progression:

Compiled results 50K and 10K microarrays (resolution 6.4K SNPs).

Relative copy number changes (CN).

Group means and standard deviations are indicated (n=46).
The peaks in the middle illustrate the relative influence of the "smoothing" of data that has been performed during data analysis (see "Methods" section in paper).

Results are not corrected for stage and grade.

Permutation analysis: P-values on the top bar refer to proportion of group label permutations in which group differences at least as extreme as pictured were found:
yellow: >0.05. orange: <0.05; red: <0.01; brown: <0.005.

Segment length analysis: p-values are indicated on the right axis and refer to the proportion of permutations finding a segment of neighbouring SNPs beyond a fixed significance threshold (p<0.01, t-test) of at least the same length as pictured on the respective chromosome.
Since significance thresholds are calculated by t-test, the segment bars differ slightly from the permutation analysis. Low bars indicate high significance. Segment length is calculated by three different methods:
blue: number of SNPs in segment; green: sum of t-values of SNPs in segment;
red: sum of t-values of SNPs in segment divided by physical length (basepairs) of segment.

This analysis was performed using the SNPTools software [http://www.birc.dk/snptools].
The data is illustrated chromosome-wise.

Add. File 2: Copy number differences between tumors with and with no subsequent progression, according to chromosomal regions.

Blue dots: Tumors with no subsequent progression.
Red dots: Tumors with subsequent progression.

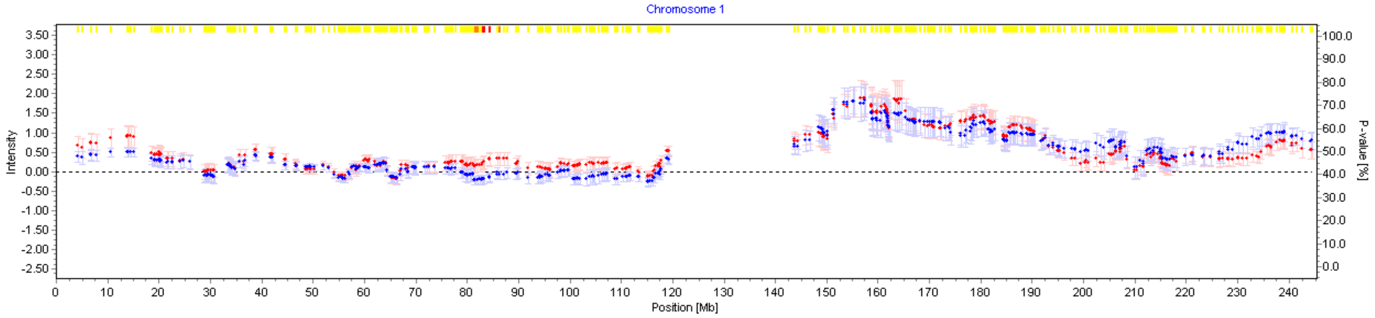

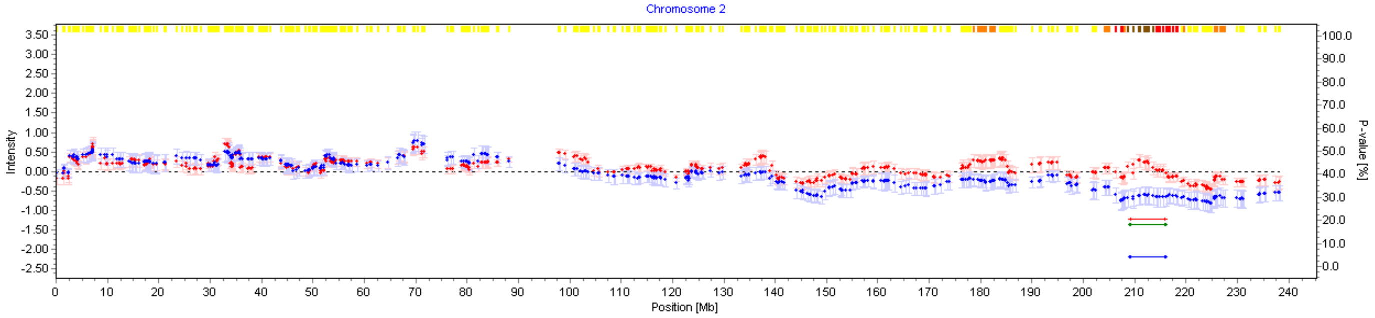

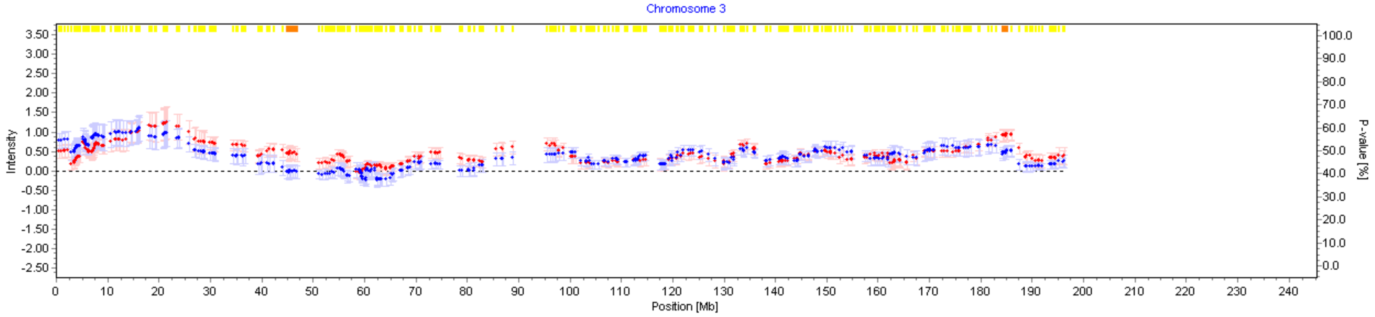

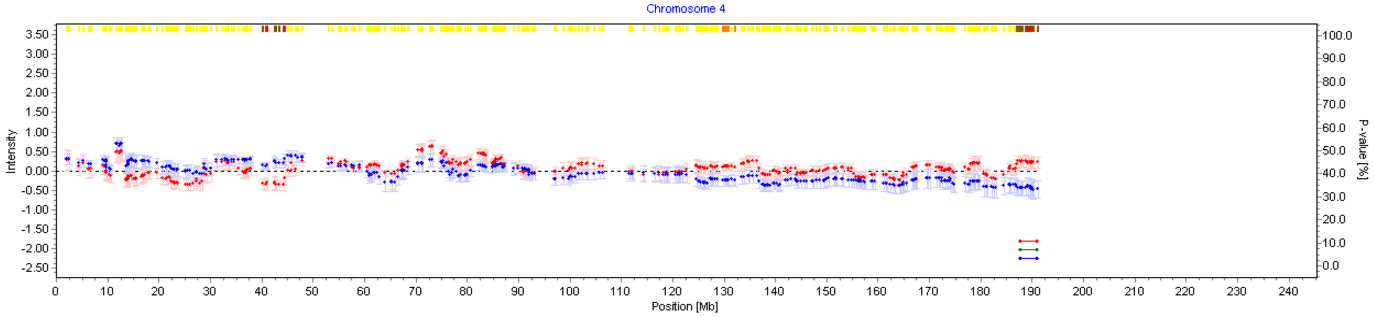

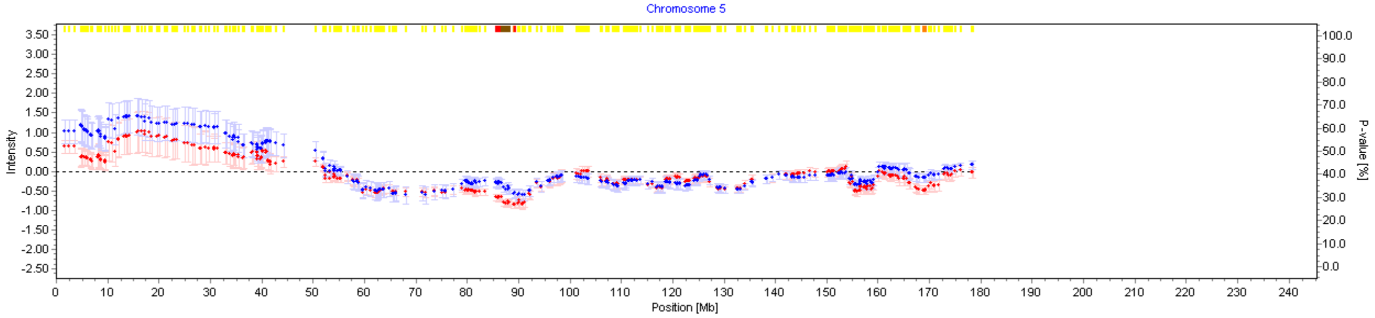

Additional File 2, continued

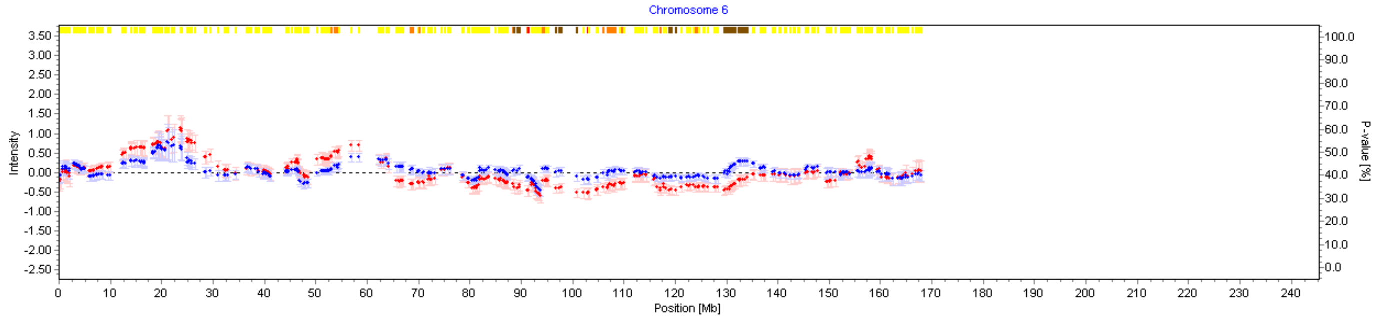

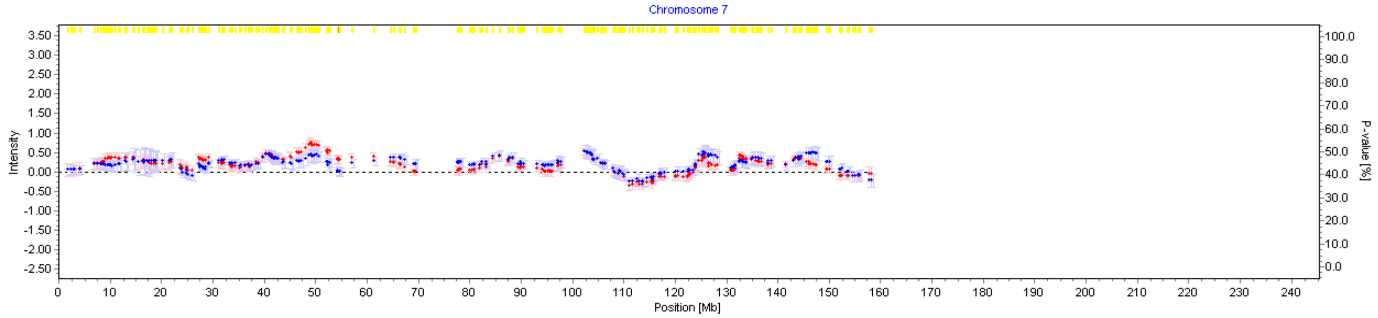

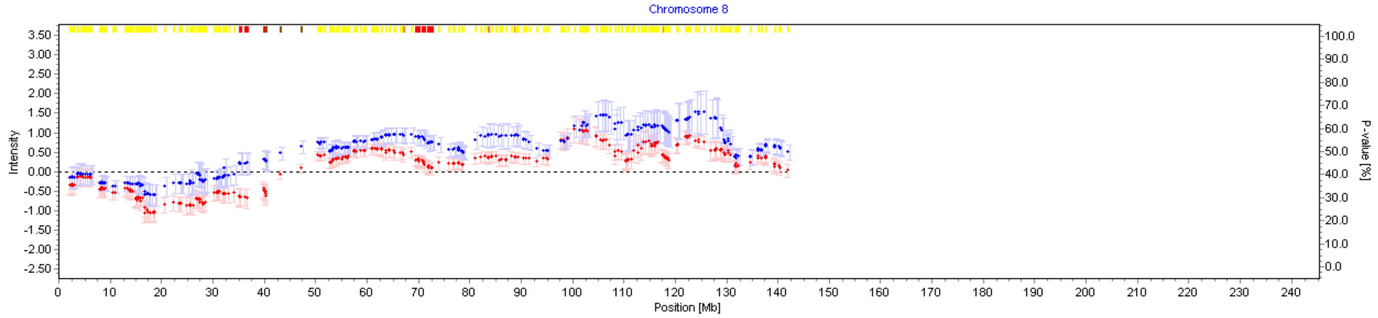

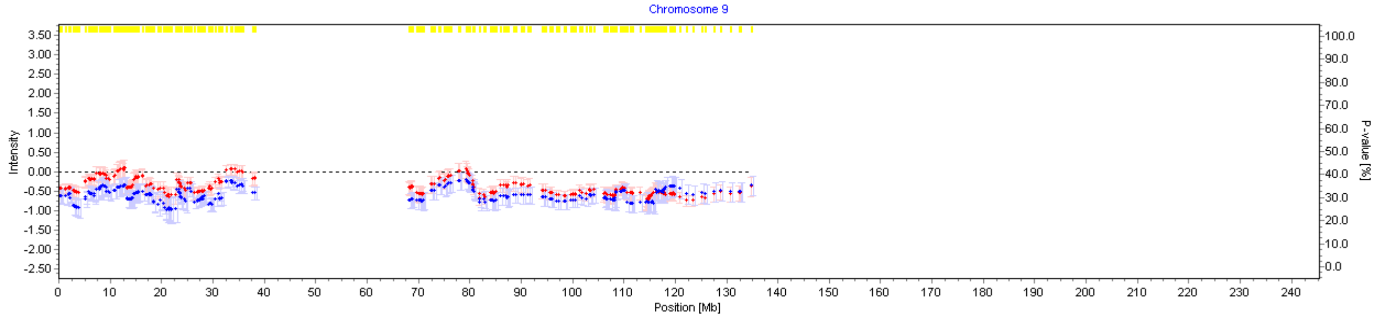

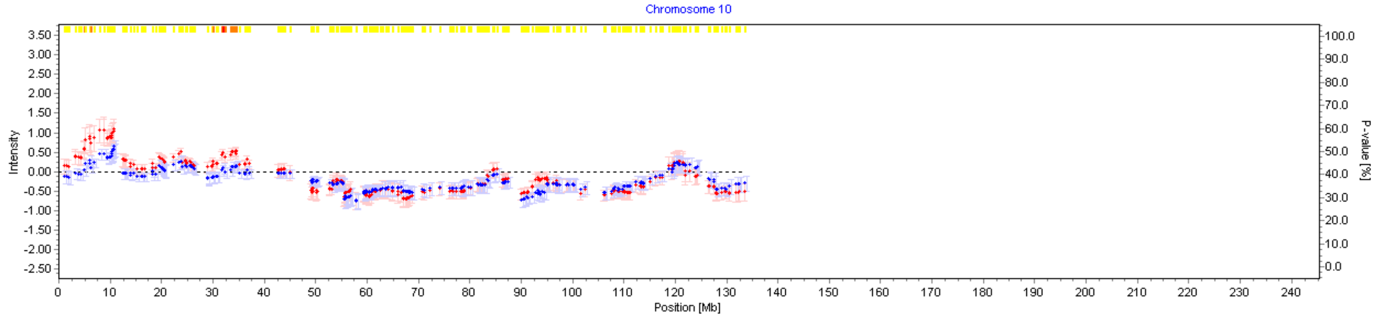

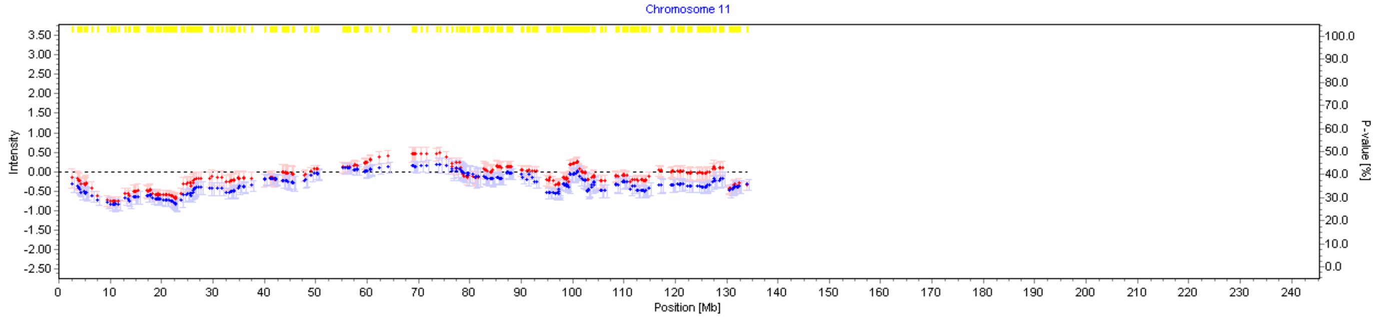

Additional File 2, continued

Chromosome 12

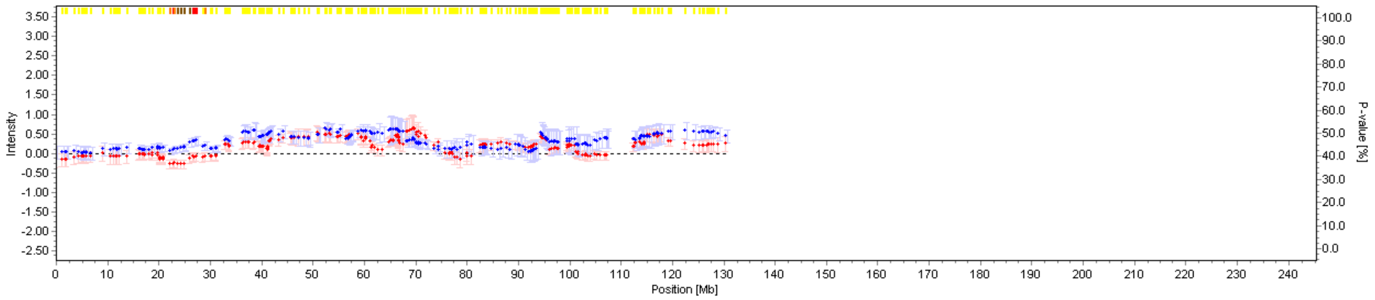

Chromosome 13

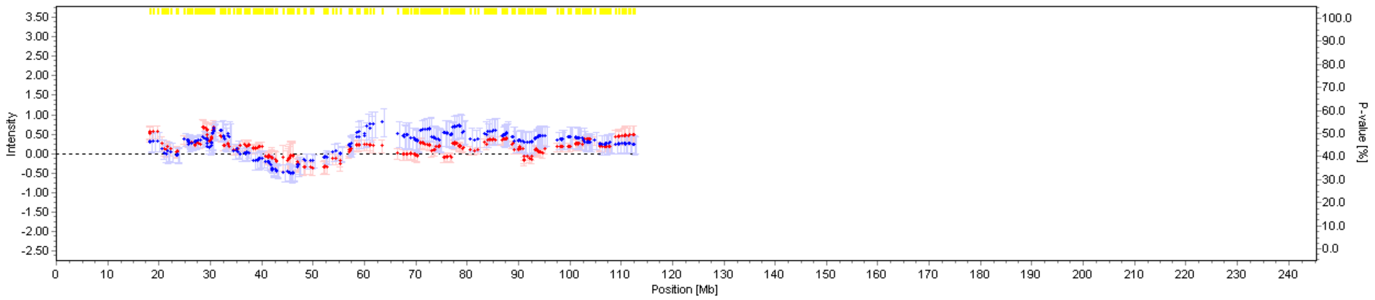

Chromosome 14

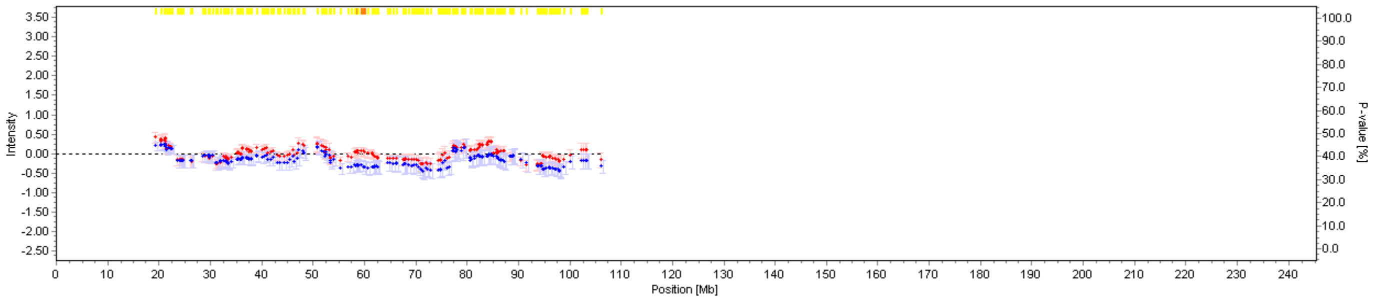

Chromosome 15

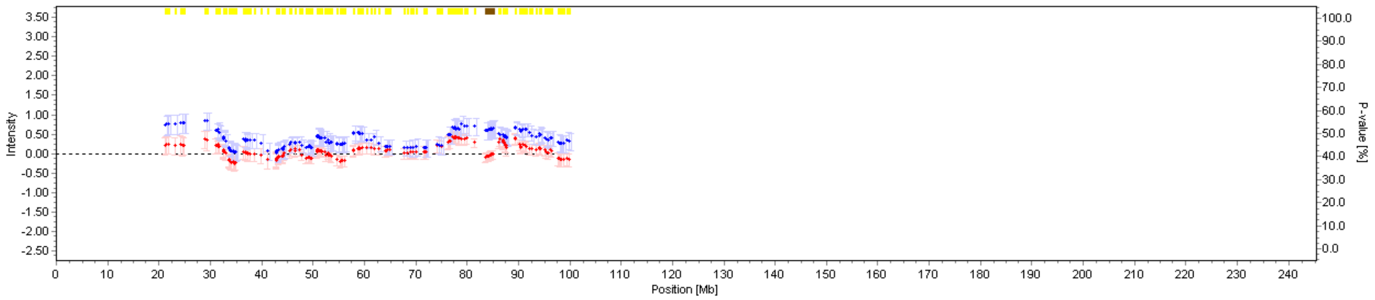

Chromosome 16

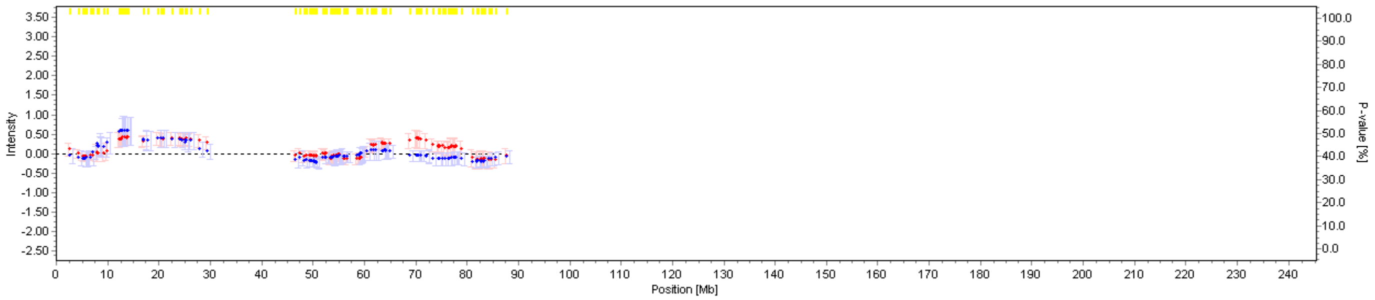

Chromosome 17

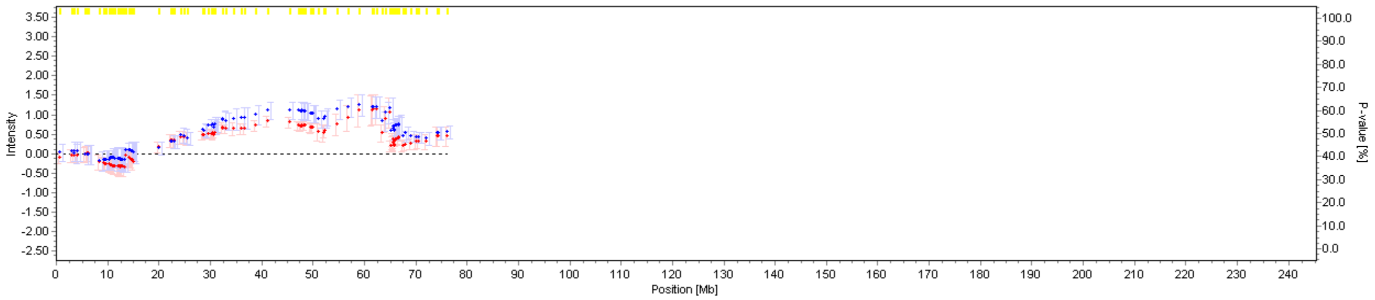

Additional File 2, continued

Chromosome 18

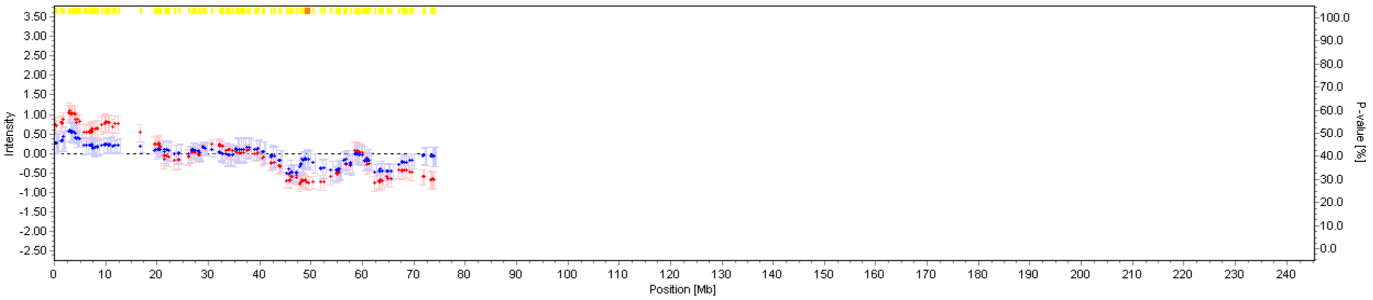

Chromosome 19

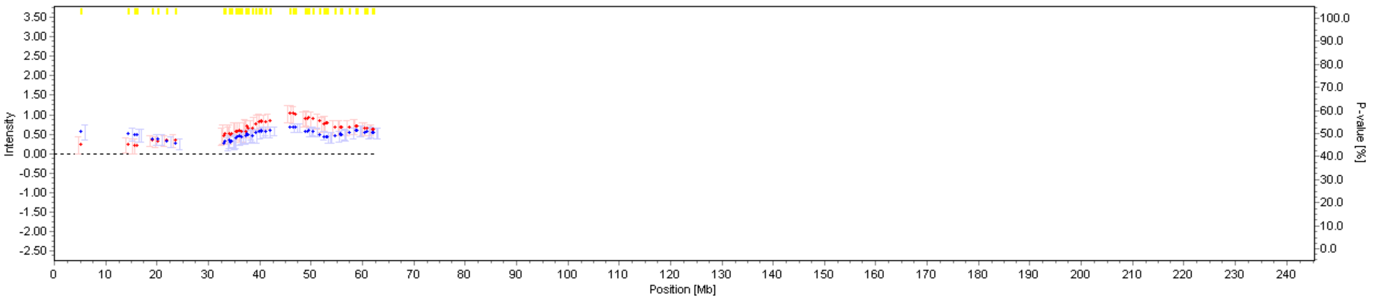

Chromosome 20

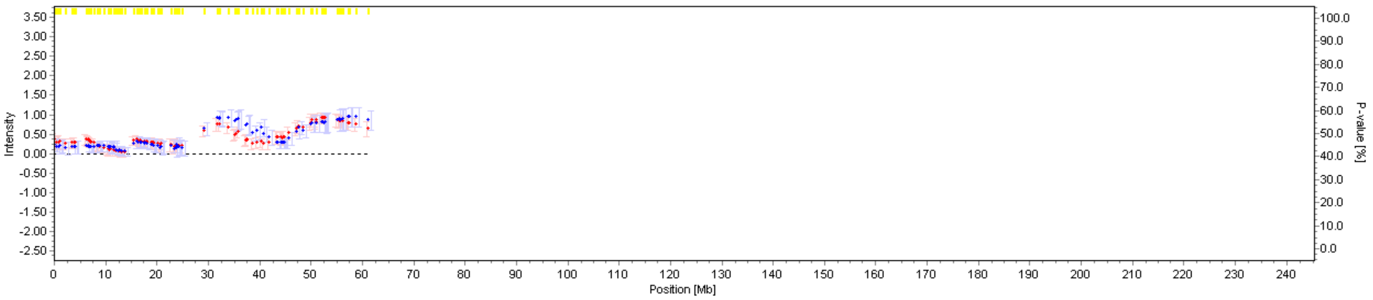

Chromosome 21

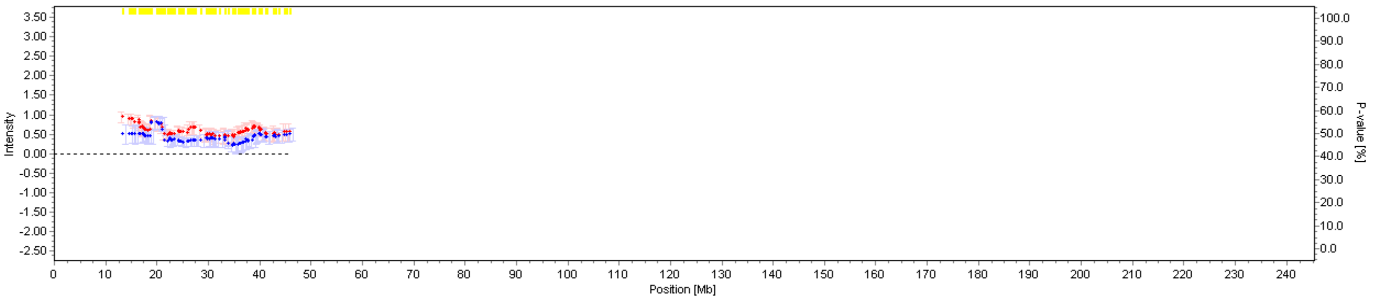

Chromosome 22

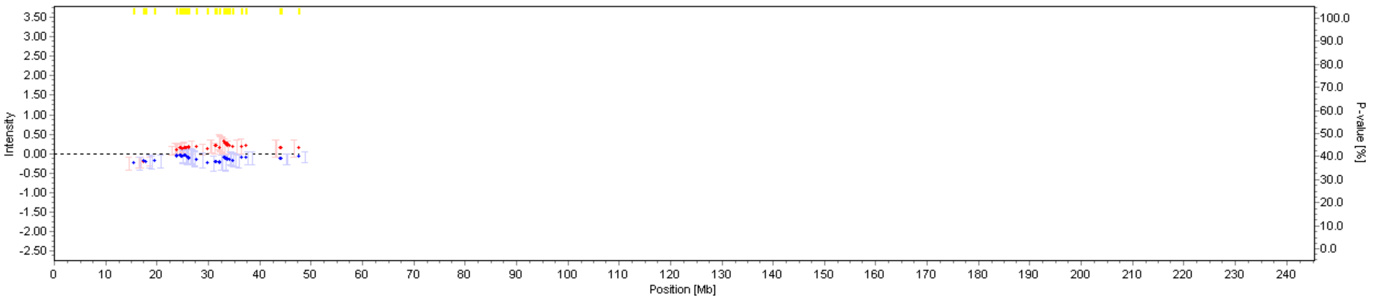

Supplement: Additional file 2 — Genome-wide copy number differences, 50 K+10 K data (6.4 K resolution). Graphical illustration of genome-wide copy number differences between tumors with and with no subsequent progression. Compiled results of all SNP microarray data (n = 46) with a resolution of 6.4 K SNPs. [file 1471-2407-9-149-S2.pdf]
