# Supplementary material for: Chromosomal imbalance in the progression of high-risk non-muscle invasive bladder cancer
Source: BMC Cancer. 2009 May 16;9:149. doi: 10.1186/1471-2407-9-149 (PMC2696467; doi:10.1186/1471-2407-9-149)

Additional File 5

Genome-wide graphical presentation and test for relative differences in chromosomal alterations between tumors with and without subsequent progression:

Results of 50K SNP microarrays only (resolution 41.4K SNPs).

Fraction of tumors with probability of LOH > 20% (LOH) (n=28).

The peaks in the middle illustrate the relative influence of the "smoothing" of data that has been performed during data analysis (see "Methods" section in paper).

Results are not corrected for stage; since stage T1 tumors are more frequent among non-progressing tumors in this series, significant alterations in non-progressing tumors are most likely related to tumor stage.

Permutation analysis: P-values on the top bar refer to proportion of group label permutations in which group differences at least as extreme as pictured were found:
yellow: >0.05. orange: <0.05; red: <0.01; brown: <0.005.

This analysis was performed using the SNPTools software [http://www.birc.dk/snptools].
Since the LOH-probability is not normally distributed, the segment analysis of these graphs could not be pictured using this software (because it uses t-statistics to calculate significance thresholds).
The data is illustrated chromosome-wise.

Add. File 5: Differences in probability of LOH between tumors with and with no subsequent progression, according to chromosomal regions.

Blue dots: Tumors with no subsequent progression (follow-up > 5 years).
Red dots: Tumors with subsequent progression.

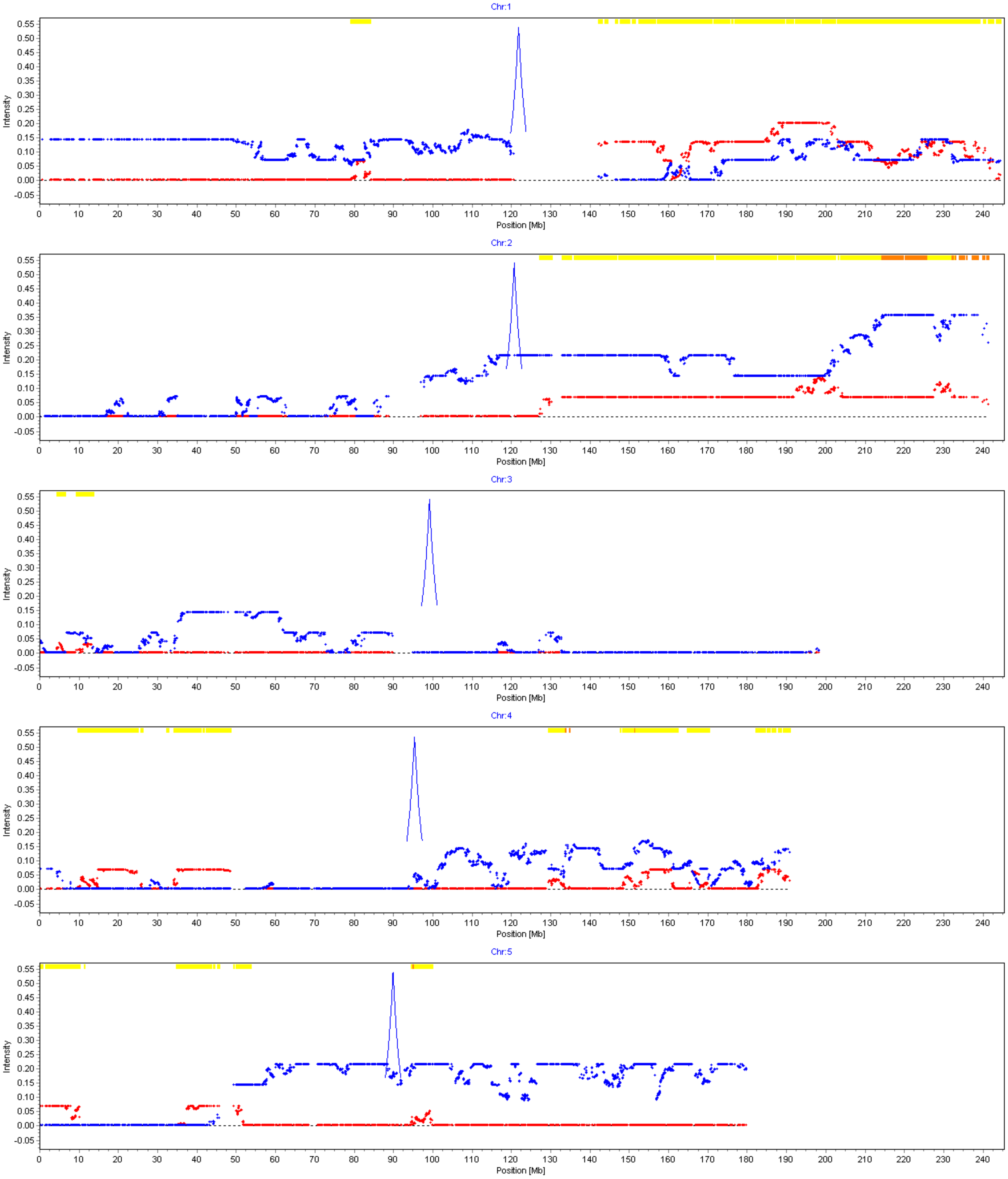

Additional File 5, continued

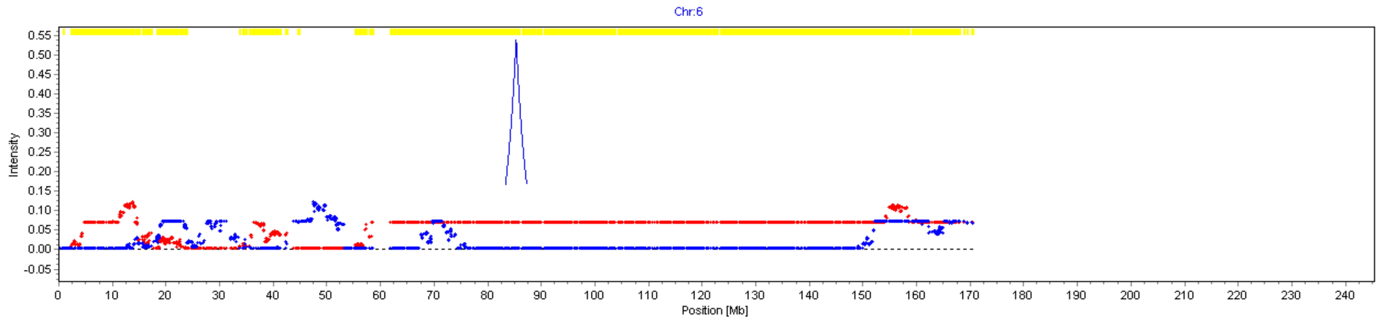

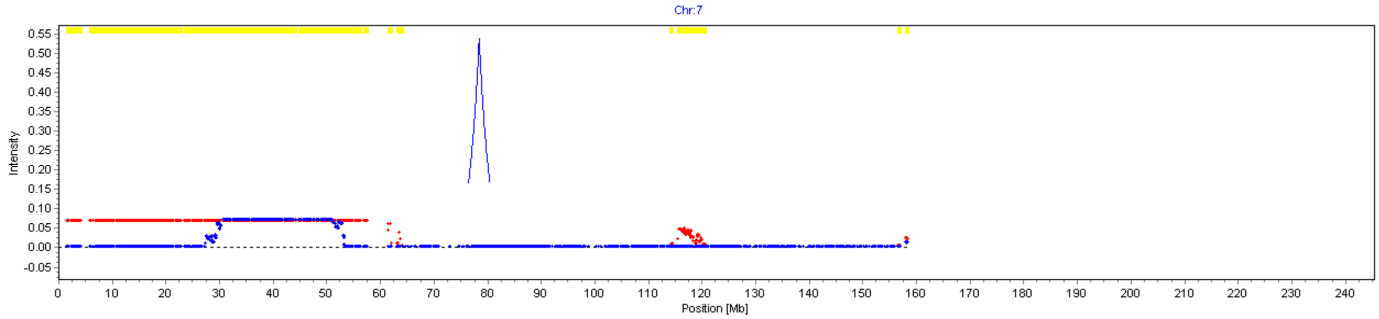

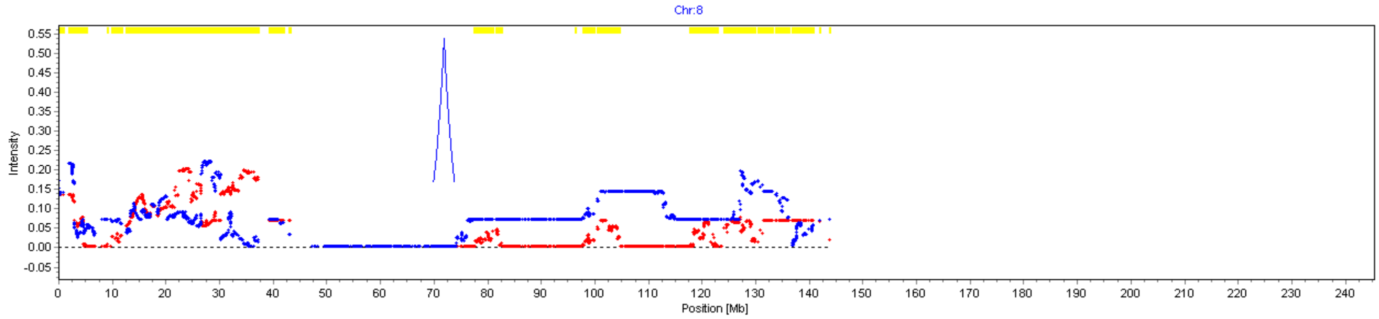

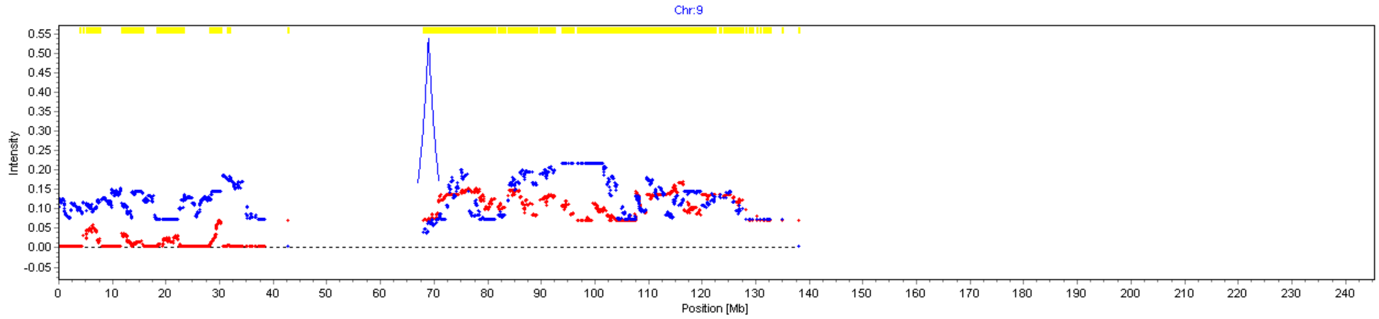

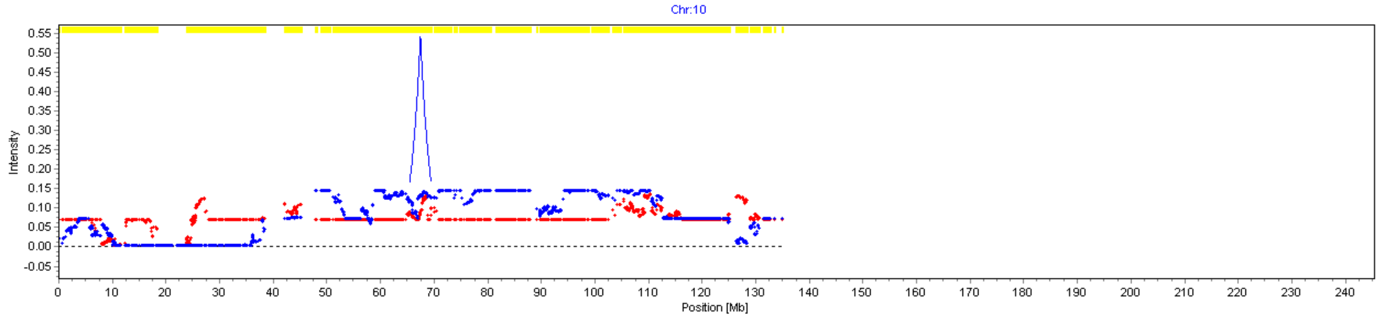

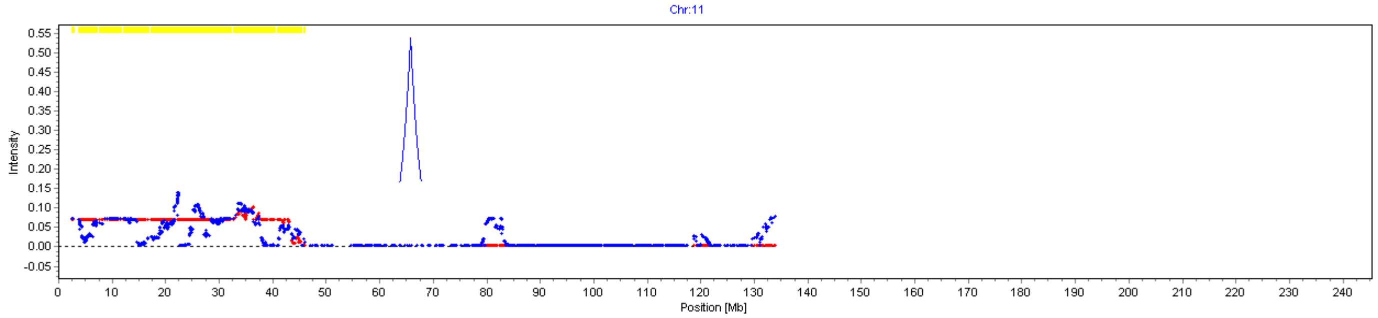

Additional File 5, continued

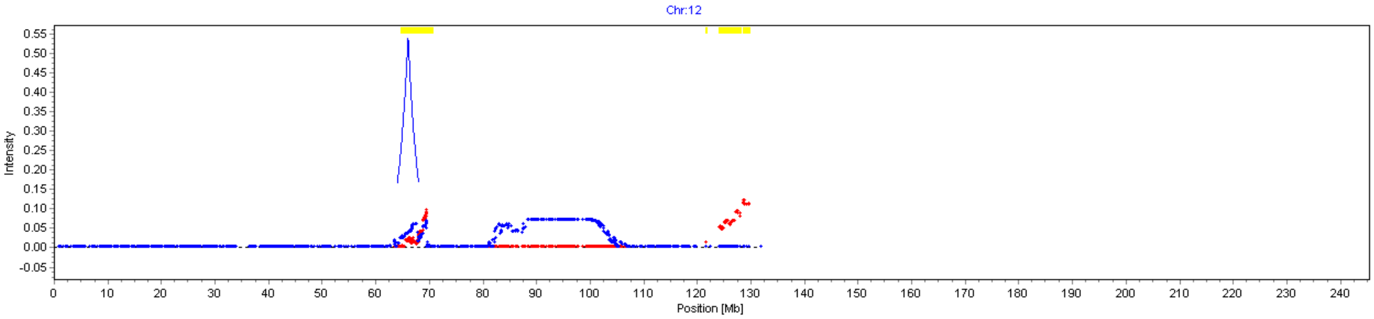

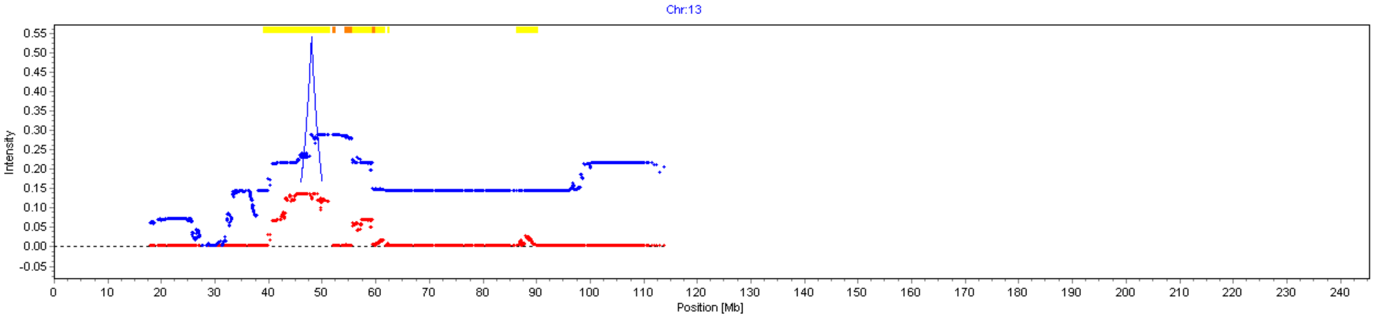

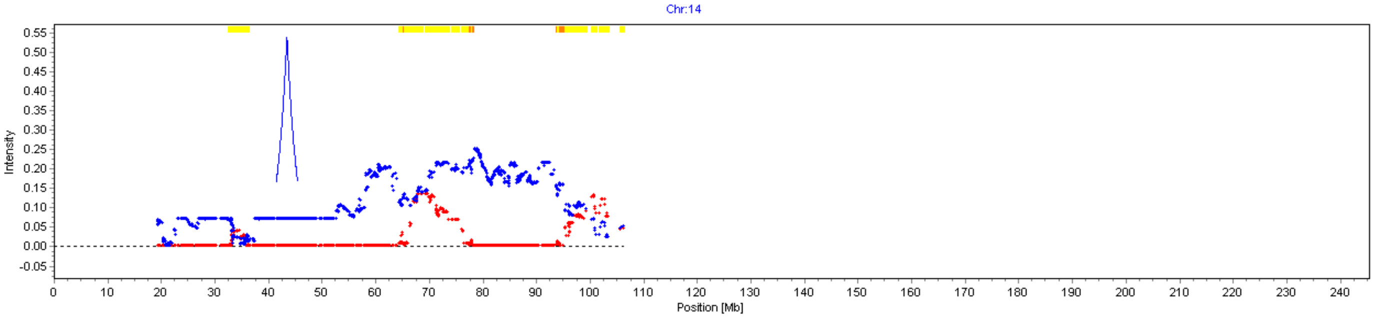

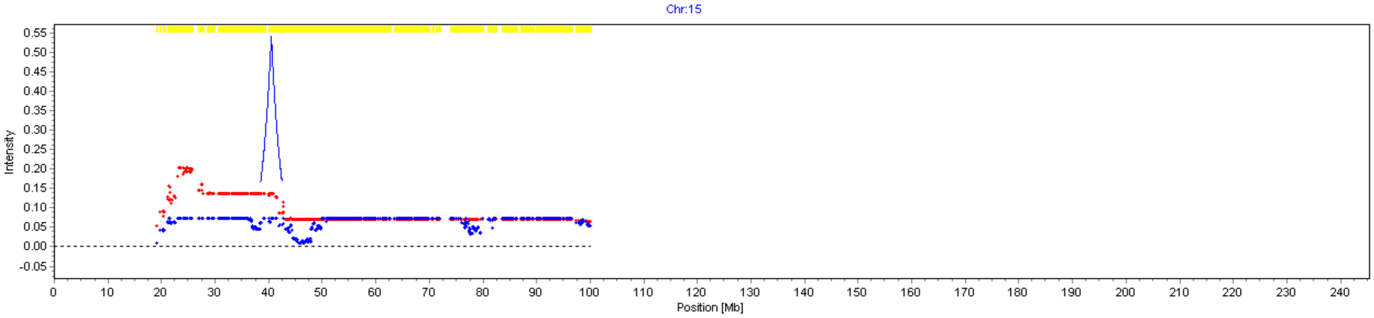

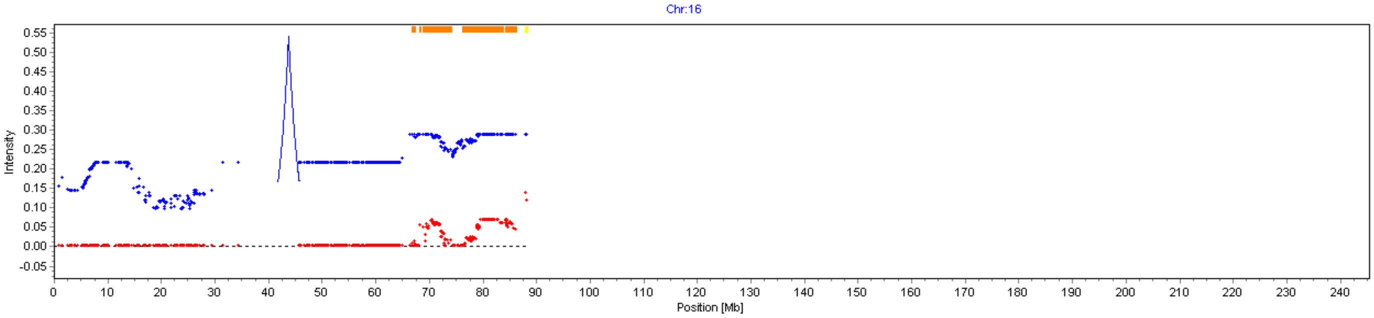

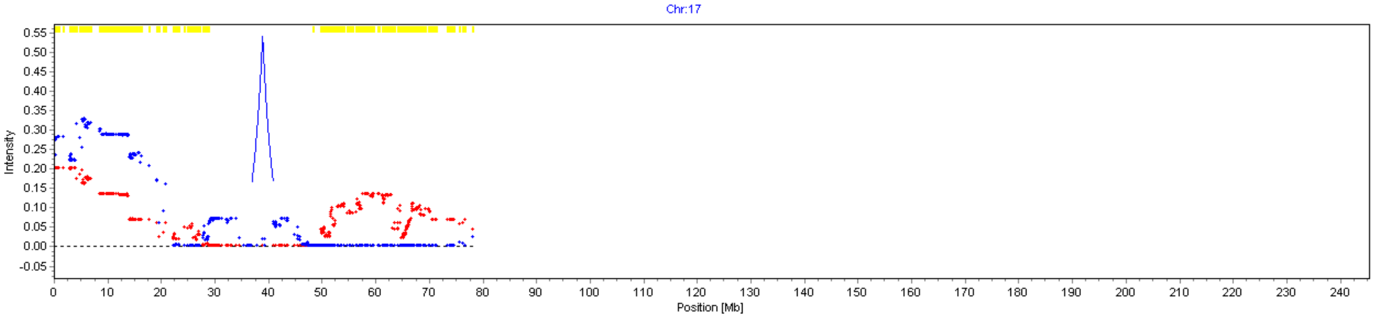

Additional File 5, continued

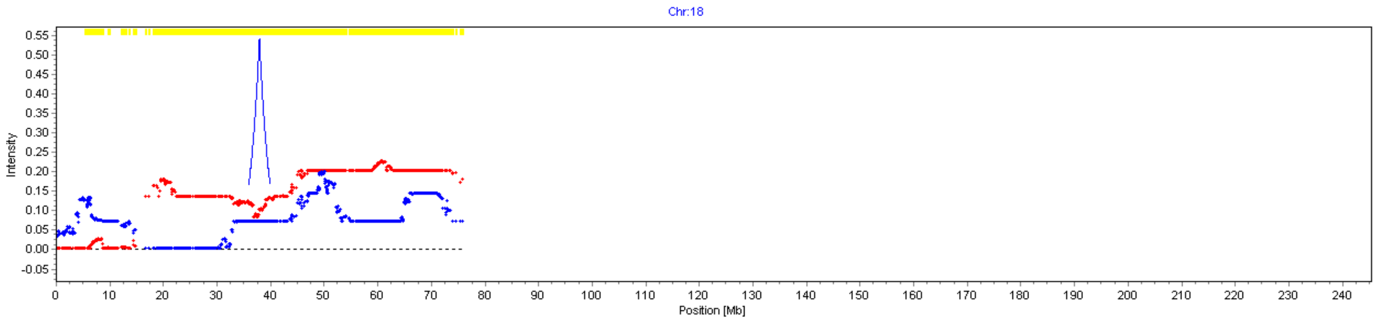

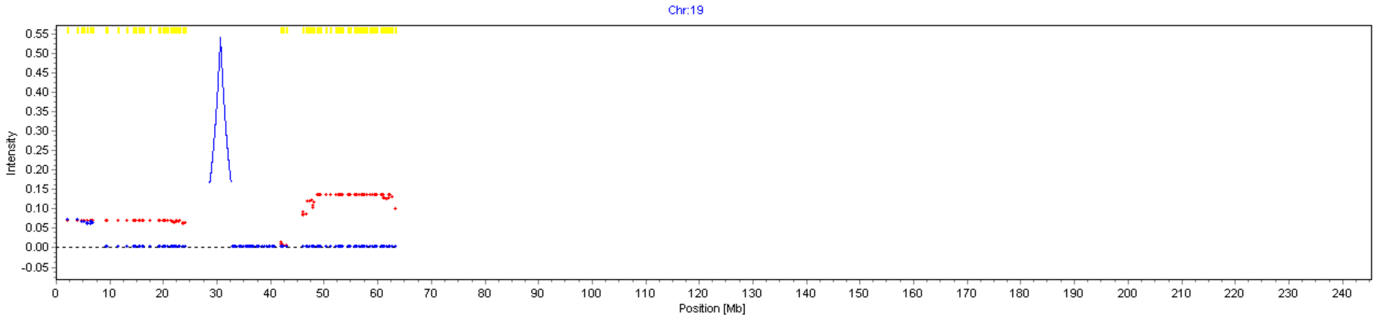

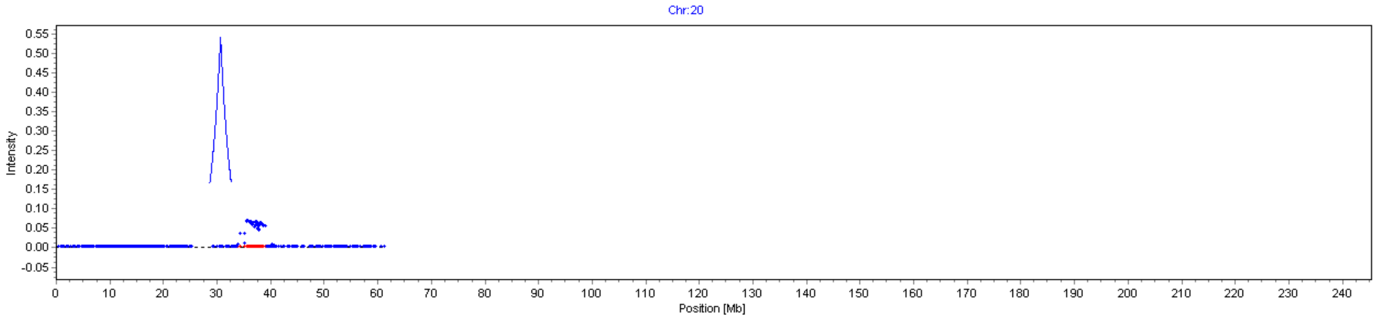

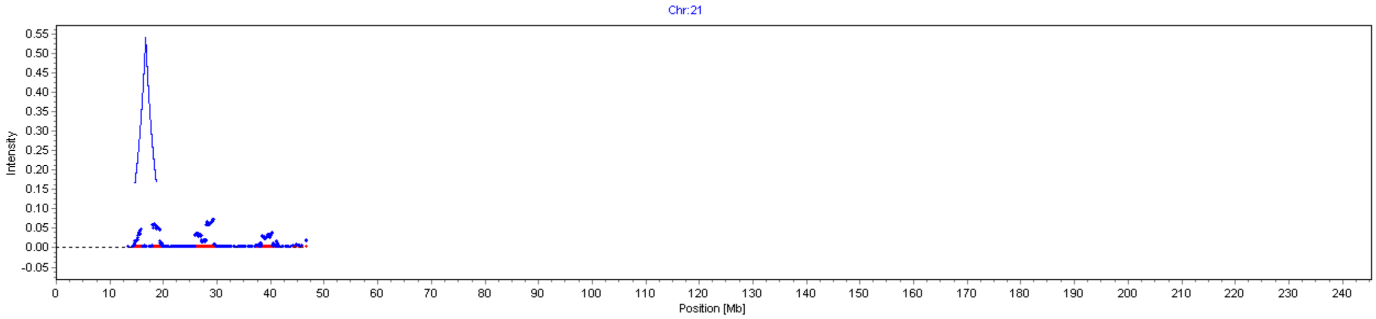

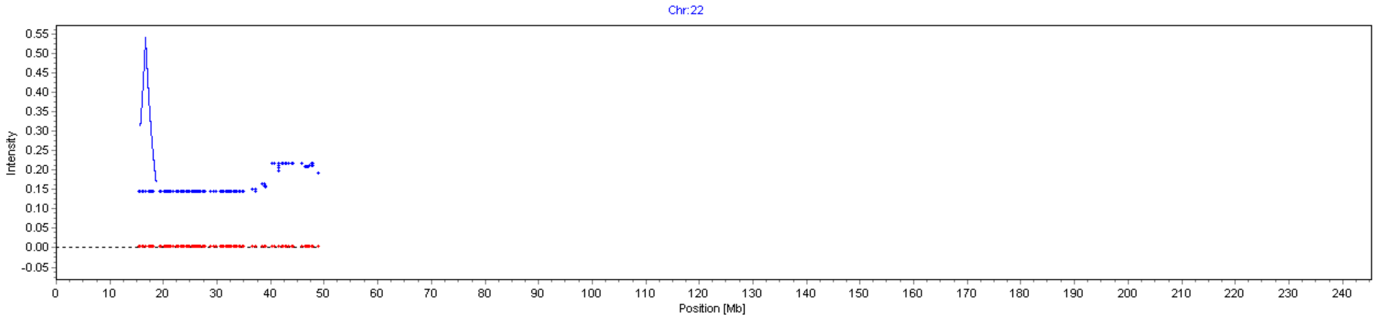

Supplement: Additional file 5 — Genome-wide differences in probability of LOH, 50 K data only. Graphical illustration of genome-wide differences in probability of LOH between tumors with and with no subsequent progression. 50 K SNP microarray data only (n = 28). [file 1471-2407-9-149-S5.pdf]
