# Supplementary material for: Chromosomal imbalance in the progression of high-risk non-muscle invasive bladder cancer
Source: BMC Cancer. 2009 May 16;9:149. doi: 10.1186/1471-2407-9-149 (PMC2696467; doi:10.1186/1471-2407-9-149)
Supplement: Additional file 6 — Selected gene loci, primer sequences and genomic annotation of the amplicons used for QPCR validation. [file 1471-2407-9-149-S6.doc]

# Add. File 6: Selected gene loci, primer sequences and genomic annotation of the amplicons used for QPCR validation.

Tm: melting temperature (determined *ex silico* using the “Primer3” software http://frodo.wi.mit.edu/cgi-bin/primer3/primer3.cgi)

| **Gene** | **Gene-ID** | Cytoband | **Primer forward** | **Tm** | **Primer reverse** | **Tm** | **Amplicon length (bases)** | **Amplicon posi­tion (NCBI Build 35.1 [hg17])** | **Amplicon position (hg18)** |
| --- | --- | --- | --- | --- | --- | --- | --- | --- | --- |
| S100A8 | 6279 | 1q21 | TGGAGAAAGCCTTGAACTCTATCA | 61,55 | AGGACACTCGGTCTCTAGCAATTT | 61,8 | 116 | chr1:150,175,956-150,176,071 | chr1:151,629,507-151,629,622 |
| FN1 | 2335 | 2q34 | AGGAAATCTGCACAACCAATGAA | 62,92 | ACCTCATCATGTGACCCATGTC | 62,04 | 87 | chr2:216,112,380-216,112,466 | chr2:215,995,119-215,995,205 |
| RAF1 | 5894 | 3p25 | TCCAGCTGCATCTCTCCTACAATA | 62,41 | AAAACACGGATAGTGTTGCTTGTC | 61,62 | 110 | chr3:12,635,039-12,635,148 | same |
| DAB2 | 1601 | 5p13 | CTGTCCATACAGAATGGCGTAAAG | 62,03 | CCTCTTCCTGGTTTGGTACTTTGT | 61,89 | 110 | chr5:39,418,493-39,418,602 | same |
| CSPG2 | 1462 | 5q14.3 | TGTCATTTTTCAACGATGCCTACT | 62,02 | TCTTTTCCATTTTTGTCCACTTCA | 61,98 | 101 | chr5:82,821,732-82,821,832 | same |
| E2F3 | 1871 | 6p22 | TGCTCAGTTTCTATGGGAAACCTT | 62,4 | AAGTTCACAAACGGTCCTTCTAGG | 62 | 107 | chr6:20,598,410-20,598,516 | same |
| SFRP1 | 6422 | 8p12-p11.1 | CTTCTACACCAAGCCACCTCAGT | 61,94 | ACCATCTTCTTGTAGCCCACGTT | 63,24 | 81 | chr8:41,285,606-41,285,686 | same |
| TUSC3 | 7991 | 8p22 | CTGAAAAAGTAGAGCAGCTGATGG | 61,71 | ATCATGGAATAGTTTCGAGGTGGT | 62,08 | 112 | chr8:15,524,970-15,525,081 | same |
| EDD1 | 51366 | 8q22 | CCAGCAGTTACCTAACATCTGCAA | 62,66 | CTTAGGAATAAGGCCCATCAAGTC | 61,43 | 92 | chr8:103,366,989-103,367,080 | same |
| MYC | 4609 | 8q24.12-q24.13 | CCAGAGGAGGAACGAGCTAA | 59,6 | TTGGACGGACAGGATGTATG | 59,4 | 130 | chr8:128,822,158-128,822,287 | same |
| CDKN2A | 1029 | 9p21 | ACTGTGTTGGAGTTTTCTGGAGTG | 61,84 | AGCTTCCCTAGTTCACAAAATGCT | 61,75 | 129 | chr9:21,957,845-21,957,973 | same |
| KLF4 | 9314 | 9q31 | CTTACTCGCCTTGCTGATTGTCTA | 62,04 | CCGAGATCCTTCTTCTTTGGATTA | 61,98 | 124 | chr9:107,331,117-107,331,240 | chr9:109,291,383-109,291,506 |
| MDM2 | 4193 | 12q14.3-q15 | GACCTAAAAATGGTTGCATTGTCC | 62,8 | GTCTACATACTGGGCAGGGCTTAT | 61,8 | 108 | chr12:67,519,751-67,519,858 | Same |
| RB1 | 5925 | 13q14.2 | TCCCATGGATTCTGAATGTG | 59,3 | GATTCCATGATTCGATGTTCAC | 59,3 | 139 | chr13:47,853,426-47,853,564 | Same |
| TP53 | 7157 | 17p13.1 | GTCCAGATGAAGCTCCCAGAAT | 61,86 | GTAGCTGCCCTGGTAGGTTTTCT | 62,12 | 146 | chr17:7,520,091-7,520,236 | Same |
